# Common haplotypes at the *CFH* locus and low-frequency variants in *CFHR2* and *CFHR5* associate with systemic FHR concentrations and age-related macular degeneration

**DOI:** 10.1016/j.ajhg.2021.06.002

**Published:** 2021-07-13

**Authors:** Laura Lorés-Motta, Anna E. van Beek, Esther Willems, Judith Zandstra, Gerard van Mierlo, Alfred Einhaus, Jean-Luc Mary, Corinne Stucki, Bjorn Bakker, Carel B. Hoyng, Sascha Fauser, Simon J. Clark, Marien I. de Jonge, Everson Nogoceke, Elod Koertvely, Ilse Jongerius, Taco W. Kuijpers, Anneke I. den Hollander

**Affiliations:** 1 Department of Ophthalmology, Donders Institute for Brain, Cognition and Behaviour, Radboud University Medical Center, Nijmegen, 6525EX, the Netherlands; 2 Roche Pharma Research and Early Development, Roche Innovation Center Basel, F. Hoffmann-La Roche Ltd, Basel, 4070, Switzerland; 3 Department of Immunopathology, Sanquin Research and Landsteiner Laboratory, Amsterdam University Medical Centre, University of Amsterdam, Amsterdam, 1066CX, the Netherlands; 4 Department of Pediatric Immunology, Rheumatology and Infectious Diseases, Emma Children’s Hospital, Amsterdam University Medical Centre, Amsterdam, 1105 AZ, the Netherlands; 5 Department of Medical Parasitology and Infection Biology, Swiss Tropical and Public Health Institute, Basel, 4051, Switzerland; 6 University of Basel, Basel, 4051, Switzerland; 7 Laboratory of Medical Immunology, Department of Laboratory Medicine, Radboud Institute for Molecular Life Sciences, Radboud University Medical Center, Nijmegen, 6525GA, the Netherlands; 8 Radboud Center for Infectious Diseases, Radboud University Medical Center, Nijmegen, 6525GA, the Netherlands; 9 Translational Metabolic Laboratory, Department of Laboratory Medicine, Radboud Institute for Molecular Life Sciences, Radboud University Medical Center, Nijmegen, 6525GA, the Netherlands; 10 University Eye Clinic, Department for Ophthalmology, University of Tübingen, 72076, Germany; 11 Institue for Ophthalmic Research, Eberhard Karls University of Tübingen, 72076, Germany; 12 Lydia Becker Institute of Immunology and Inflammation, Faculty of Biology, Medicine and Health, University of Manchester, M139PL, United Kingdom; 13 Department of Blood Cell Research, Sanquin Research and Landsteiner Laboratory, Amsterdam University Medical Center, University of Amsterdam, Amsterdam, 1066CX, the Netherlands; 14 Department of Human Genetics, Donders Institute for Brain, Cognition and Behaviour, Radboud University Medical Centre, 6525GA, the Netherlands

**Keywords:** age-related macular degeneration, AMD, complement system, CFH, CFHR2, CFHR5, complement Factor H, complement-factor-H-related

## Abstract

Age-related macular degeneration (AMD) is the principal cause of blindness in the elderly population. A strong effect on AMD risk has been reported for genetic variants at the *CFH* locus, encompassing complement factor H (*CFH*) and the complement-factor-H-related (CFHR) genes, but the underlying mechanisms are not fully understood. We aimed to dissect the role of factor H (FH) and FH-related (FHR) proteins in AMD in a cohort of 202 controls and 216 individuals with AMD. We detected elevated systemic levels of FHR-1 (p = 1.84 × 10^−6^), FHR-2 (p = 1.47 × 10^−4^), FHR-3 (p = 1.05 × 10^−5^) and FHR-4A (p = 1.22 × 10^−2^) in AMD, whereas FH concentrations remained unchanged. Common AMD genetic variants and haplotypes at the *CFH* locus strongly associated with FHR protein concentrations (e.g., FH p.Tyr402His and FHR-2 concentrations, p = 3.68 × 10^−17^), whereas the association with FH concentrations was limited. Furthermore, in an International AMD Genomics Consortium cohort of 17,596 controls and 15,894 individuals with AMD, we found that low-frequency and rare protein-altering *CFHR2* and *CFHR5* variants associated with AMD independently of all previously reported genome-wide association study (GWAS) signals (p = 5.03 × 10^−3^ and p = 2.81 × 10^−6^, respectively). Low-frequency variants in *CFHR2* and *CFHR5* led to reduced or absent FHR-2 and FHR-5 concentrations (e.g., p.Cys72Tyr in *CFHR2* and FHR-2, p = 2.46 × 10^−16^). Finally, we showed localization of FHR-2 and FHR-5 in the choriocapillaris and in drusen. Our study identifies FHR proteins as key proteins in the AMD disease mechanism. Consequently, therapies that modulate FHR proteins might be effective for treating or preventing progression of AMD. Such therapies could target specific individuals with AMD on the basis of their genotypes at the *CFH* locus.

## Introduction

Age-related macular degeneration (AMD) is responsible for the majority of blindness occurring among the elderly in the Western world and is the third most common cause of severe visual impairment worldwide.^1^^,^^2^ The prevalence increases dramatically with age^3^ and, with an aging society, the number of affected individuals is expected to rise from 196 million to 288 million by 2040.^4^ The early stage of AMD is characterized by the appearance of drusen under the retina.^5^ Drusen contain inflammatory factors, suggesting a local chronic inflammatory state.^6^ Individuals with AMD can progress to an advanced stage of disease in which vision loss occurs.^7^ Advanced AMD can be classified as two types: (1) geographic atrophy, involving degeneration of the choriocapillaris, retinal pigment epithelium (RPE) cells and photoreceptors; and (2) choroidal neovascularization, in which abnormal sprouting of blood vessels from the choriocapillaris occurs.^8^ Therapeutic options to date are limited to the neovascular form and have variable effectiveness, leaving a vast number of individuals with AMD untreated or with a progressing disease.^9^

AMD is a multifactorial disease in which genetic factors play a significant role, explaining 46% to 71% of the variation in the overall severity.^10^ Identification of these genetic factors and understanding how they exert their effect on AMD can help disentangle the AMD disease mechanisms and lead to improved therapeutic options. The first major susceptibility locus for AMD was identified on chromosome 1 at the 1q31.3 locus, where the rs1061170 (p.Tyr402His) variant in complement factor H (*CFH*) was strongly associated with an increased risk for AMD.^11^, ^12^, ^13^, ^14^ This finding soon implicated factor H (FH), a major regulator of the alternative pathway of the complement cascade, and its short splice variant FH like-1 (FHL-1) in the development of AMD. More recently, the International AMD Genomics Consortium (IAMDGC) performed a large genome-wide association study (GWAS) on advanced AMD. This study included 17,832 controls and 16,144 individuals with advanced AMD and identified 52 independent genome-wide significant signals at 34 genomic loci.^15^ Out of these 52 signals, a total of eight are located at an extended *CFH* locus, encompassing *KCNT2*, *CFH*, *CFHR1*, *CFHR2*, *CFHR3*, *CFHR4*, and *CFHR5*. The lead variants of these signals include only one *CFH* missense variant, rs121913059 (p.Arg1210Cys), which exerts a very strong effect on AMD (odds ratio [OR] = 20.28). The remaining variants are synonymous or non-coding, and one, rs570618, is in high linkage disequilibrium (LD) with the rs1061170 (p.Tyr402His) variant in *CFH* (r^2^ = 0.99, phase 3 v5 of the 1000 Genomes Project). The p.Arg1210Cys and p.Tyr402His variants are reported to affect the binding properties of FH (and FHL-1 in the case of p.Tyr402His), resulting in reduced function of the protein, which in turn would lead to a more active state of the complement system and local chronic inflammation.^16^, ^17^, ^18^, ^19^, ^20^ The underlying effects of the other AMD-associated signals at this locus are, however, not known.

Several lines of evidence suggest that genetic variants at the extended *CFH* locus might affect the CFHR genes. The complement FH-related (FHR) proteins are thought to compete with FH and fine tune complement regulation, but their function is not yet fully understood.^21^ A common deletion spanning *CFHR3* and *CFHR1* (CNP147, 86.3 kb) and a rare deletion spanning *CFHR1* and *CFHR4* (CNP148, 122 kb) are both associated with a protective effect for AMD.^22^, ^23^, ^24^, ^25^, ^26^, ^27^, ^28^, ^29^ Additionally, systemic FHR-4 concentrations have been recently found to be elevated in individuals with AMD and to be associated with AMD genetic variants.^30^ These findings suggest that FHR proteins might be involved in AMD, underlying the effect of the unexplained signals at the extended *CFH* locus, yet a comprehensive analysis of all five FHR proteins and the eight AMD genetic variants has not been performed to date.

In this study we aimed to dissect the role of FH and all five FHR proteins encoded by genes at the extended *CFH* locus in AMD. We measured serum concentrations of FH and the FHR proteins in individuals with advanced AMD and controls by using highly specific ELISA and assessed associations with AMD disease and with common variants and haplotypes at the extended *CFH* locus. In addition, we assessed the association of low-frequency and rare protein-altering variants in the *CFH* and CFHR genes with AMD and with systemic protein concentrations. Finally, we examined the localization of the FHR proteins at the local AMD disease site in the eye.

## Material and methods

### Study cohort

Individuals included in this study were retrieved from the European Genetic Database (EUGENDA), a database created for the study of AMD. EUGENDA encompasses clinical and molecular information collected at the Radboud University Medical Center, Nijmegen, the Netherlands, and at the University Hospital of Cologne, Cologne, Germany. Certified graders determined the AMD and control status of each individual included in the study by using multimodal image grading according to the standard protocol of the Cologne Image Reading Center (as described in Heesterbeek et al., 2020; Table S1).^31^ Controls included in this study were older than 65 years, and individuals with AMD were older than 60 years. All participants were ascertained to be of European descent via genome-wide array genotyping (see below). Information on age and sex were obtained from standardized interviewer-assisted questionnaires, and information on sex was confirmed with genetic data (see below).

Written informed consent was obtained from all study participants regarding clinical examination, epidemiological data collection, and blood measurements, as well as genetic analyses. This study adhered to the tenets of the Declaration of Helsinki (7^th^ revision), and ethical approval was obtained from the local ethics committees (Arnhem-Nijmegen Commissie Mensgebonden Onderzoek (CMO) and Ethics Commission of Cologne University’s Faculty of Medicine).

### Protein measurements

Serum concentrations of FH, the FHR proteins, and the homo- and heterodimers of FHR-1 and FHR-2 were measured in serum samples of the EUGENDA AMD case-control cohort. In total, 418 serum samples of 202 controls and 216 individuals with advanced AMD were included in the study. Serum samples were obtained via a standard coagulation and centrifugation protocol and were stored at –80°C within 1 h. Samples were not thawed before being used for this study. Serum concentrations of FH and FHR proteins were determined in two batches of 130 and 288 samples, respectively (Table S1), by in-house developed ELISAs.^32^, ^33^, ^34^ For specific measurements of FH, an in-house generated, monospecific mouse monoclonal antibody (mAb) directed against CCP domains 16/17 of FH (anti-FH.16, Sanquin Research, Amsterdam, the Netherlands) was used as capture mAb, and polyclonal goat anti-human FH (Quidel, San Diego, CA, USA), conjugated with HRP in house, was used as detection Ab.^35^ FHR-1/1 homodimers and FHR-1/2 heterodimers were captured by anti-FH.02 (Sanquin Research, directed against CCP20 of FH and CCP5 of FHR-1) and detected with biotinylated anti-FH.02 or anti-FHR-2 (monospecific for FHR-2, clone MAB5484, R&D Systems), respectively.^35^ FHR-2/2 homodimer concentrations and total concentrations of both FHR-1 and FHR-2 were calculated on the basis of the observed concentrations of FHR-1/1 and FHR-1/2 dimers and in consideration of the molecular weights of 39.5 kDa for FHR-1 (average of 37 and 42 kDa) and 26.5 kDa for FHR-2 (average of 24 and 29 kDa), respectively.^36^, ^37^, ^38^, ^39^ Hence, in the 14 cases where FHR-1 protein was lacking in serum, FHR-2/2 and total FHR-2 concentrations could not be determined. For measurements of FHR-3, the in-house mAb anti-FHR-3.1 (directed against FHR-3 and FHR-4A, Sanquin Research) was used as a capture mAb, and biotinylated mAb anti-FHR-3.4 (directed against FHR-3 and FH, Sanquin Research) was used as a detecting mAb. FHR-4A was captured by monospecific mAb anti-FHR-4A.04 (directed against CCP5) and detected by biotinylated rabbit anti-human FHR-3 antiserum (Sanquin Research). Lastly, FHR-5 was measured with the monospecific anti-FHR-5.1 as a capture mAb and the monospecific biotinylated anti-FHR-5.4 as a detection mAb (both from Sanquin Research). All assays included a standard curve of normal human serum pool (>400 donors) and two control sera to ensure limited inter-assay variation. Sera were tested as two independent duplicates (on separate plates) and measured in two dilutions.

### Genotypic data

Except for one individual with advanced AMD and one control individual, all individuals with measured FH and FHR serum concentrations were part of the IAMDGC GWAS and had been genotyped with a custom-modified Illumina HumanCoreExome array at the Centre for Inherited Disease Research (CIDR).^15^ Genotype calling, ancestry, and sex assessment, as well as genotype imputation via the 1000 Genomes Project reference panel, were performed by the IAMDGC as previously described.^15^

### Statistical analysis

#### Association of FH and FHR concentrations with advanced AMD

A total of 202 controls and 216 individuals with advanced AMD and ELISA-based FH and FHR measurements were included in the analysis. A log +1 transformation and a standardization were applied to the concentrations of FH and FHR proteins. Association analyses of FH and FHR protein concentrations with advanced AMD were performed by means of a logistic regression using the ‘glm’ function of R; adjustments were made for age, sex, measuring batch (first or second), and sampling cohort (University Hospital of Cologne or Radboud University Medical Center in Nijmegen).^40^ The resulting ORs reflect the change in odds for advanced AMD per 1 SD increase of each log-transformed +1 protein concentration.

To assess relations between FH family proteins, we performed Pearson correlations separately for the AMD and control groups. Analyses were carried out with the “cor” and ‘cor.mtest’ functions of R the package “corrplot” version 0.84 and were plotted with the “corrplot” function.^41^ The correlation coefficients in the AMD and control groups were compared via the “r.test.” We found strong correlations between FH and FHR proteins (Figure S1), and therefore we corrected for multiple testing by applying a false-discovery rate (FDR) Benjamini-Hochberg procedure.^42^ The threshold for statistical significance was then defined as FDR adjusted p value < 0.05.

#### Association of FH and FHR concentrations with AMD variants and haplotypes

A total of 201 controls and 215 individuals with advanced AMD had genetic data available and were included in the analysis. Association analysis of FH and FHR concentrations with the eight AMD genetic variants (coded as 0, 1, or 2) at the extended *CFH* locus was performed with general linear models (“glm’” function of R) adjusted for age, sex, measuring batch, sampling cohort, and AMD status.^15^ Resulting betas reflect the allele effect on log-transformed +1 and standardized protein concentrations. Haplotype analyses were carried out similarly on haplotypes with a frequency >1% via the haplo.glm function of the R library “haplo.stats” (version 1.7.7),^43^ which allows for ambiguous haplotypes.

#### GWASs on FH and FHR protein concentrations

GWASs on FH, total FHR-1, total FHR-2, FHR-3, FHR-4A, and FHR-5 concentrations were performed on the entire study cohort through the use of linear models as implemented in EPACTS (“q.lm” function). The log +1 transformed and standardized protein concentrations were used as dependent variables, and the analysis was adjusted for age, sex, measuring batch, sampling cohort, AMD status, and ten ancestry principal components. Imputation quality control was performed as previously described, and genotype dosages were used for analysis of imputed variants.^15^ For the eight AMD-associated variants,^15^ we additionally performed comparable analyses without adjusting for AMD status and by including only controls. Comparable effect estimates were found for the associated variants, suggesting that adding AMD status as a covariate did not bias the estimation of the genetic effects on FH and FHR concentrations (Table S2).

Manhattan and quantile-quantile plots were generated with the “qqman” R package (version 0.1.4).^40^^,^^44^ In order to assess whether more than one genome-wide-significant signal was present at the extended *CFH* locus in each of the GWASs, we repeated the analysis conditioning on the lead variant(s) at this locus by adding the index variant(s) as covariates to the model until we found no additional genome-wide associations.

#### Low-frequency and rare-variant analysis of *CFH* and *CFHR* genes via the IAMDGC dataset

For the association analysis of low-frequency and rare variants with AMD, genotypes at the extended *CFH* locus were extracted from the IAMDGC dataset, which included 17,596 controls and 15,894 individuals with AMD (a total of 33,488 individuals). Gene-based tests were performed with SKAT-O as implemented in EPACTS, and the ENCODE v14 annotation was used.^45^ Protein-altering variants defined as missense, nonsense, and frameshift and as affecting the canonical splice sites with a MAF < 0.05 were included in the analyses. A threshold on imputation quality of >0.8 was set for the low-frequency and rare variants. The SKAT-O gene-based tests of low-frequency and rare variants were adjusted for the first two ancestry principal components and source of DNA (whole-blood or whole-genome amplified DNA). Furthermore, and in order to confirm that the associations were not driven by known AMD-associated variants in the same locus, we performed the same analysis by adding as covariates the index variants of the signals that have been previously reported to be associated with AMD at this locus (rs187328863, rs148553336, rs570618, rs10922109, rs35292876, rs121913059, rs61818925, and rs191281603), that is, a locus-wide conditioned analysis.^15^ We performed this analysis in two steps: first we adjusted only for the common index variants (rs570618, rs10922109, rs61818925), and then we adjusted for all eight index variants. We also performed sensitivity analyses by adjusting for seven additional principal components and including only variants with a PHRED-CADD score >10. We applied multiple testing correction to the gene-based tests by using the Bonferroni procedure.

#### Association of low-frequency and rare variants in *CFH*, *CFHR2,* and *CFHR5* with systemic FH, FHR-2, and FHR-5 protein concentrations

The association of low-frequency and rare variants identified in the gene-based test (see above) with (log +1 transformed and standardized) FH and FHR concentrations was modeled with adjustment for age, sex, measuring batch, sampling cohort, and AMD status, as well as for the eight variants that had previously been associated with AMD (locus-wide conditioning).^15^ All the association analyses were performed with the “glm” R function.^40^

### Immunocytochemistry of human retinas

Human donor eyes were obtained from Discovery Life Sciences. Eyes were collected within 12 h of death and transected behind the limbus so that the anterior part including the lens was removed. The posterior eyecup and the globe were immediately fixed in 4% paraformaldehyde solution for 24 h. Next, the tissue was transferred into PBS for 48 h. The vitreous was carefully removed, and the eyecup was cryopreserved in 30% sucrose overnight. The tissue was embedded in Tissue-Tek O.C.T. Compound (Sakura Finetek Europe B.V., Alphen aan den Rijn, the Netherlands). 8 μm cryosections were cut with a CryoStar NX70 Cryostat (Thermo Scientific).

The immunostaining was performed with the VENTANA DISCOVERY ULTRA automated staining system (Ventana Medical Systems). Sections from three different donors were incubated with the primary antibodies (anti-FHR-2.1, Sanquin (2.0 mg/mL), diluted [dil.] 1:250; anti-FHR-5.4, Sanquin (2.7 mg/mL), dil. 1:800) for 30 min at 37°C. The antibody anti-FHR-2.1 was generated against FHR-2 but also recognizes FHR-1. Anti-FHR-5.4 is monospecific for FHR-5. After thorough washing, sections were incubated with anti-mouse secondary antibodies (OmniMap anti-mouse HRP detection system, Roche Diagnostics) for 15 min at 37°C, followed by incubation with the DISCOVERY Purple chromogen for another 15 min. Sections were counterstained with VENTANA HE 600 Blueing in conjunction with VENTANA HE 600 Hematoxylin for 4 min and mounted with Kaiser’s glycerol gelatin (Merck). Images were captured by a VS120 Olympus slide scanner and processed by VS-ASW L100 imaging software (version 2.9., Olympus). The results for FHR-2 were confirmed with a monospecific antibody clone detecting FHR-2 (MAB5484, R&D Systems) and frozen sections from three donors as described above. All tissues were acquired with consent of the donor or donor family in accordance with the principles outlined in the Declaration of Helsinki.

## Results

### Systemic concentrations of FHR-1, FHR-2, FHR-3, and FHR-4A are higher in individuals with advanced AMD than in controls

The five CFHR genes, located downstream of *CFH,* encode FHR-1, FHR-2, FHR-3, FHR-4A, FHR-4B, and FHR-5; FHR-4A is the only splice variant of *CFHR4* found in circulation.^34^ FHR-1, FHR-2, and FHR-5 are present as homodimers, and FHR-1 and FHR-2 are also able to form heterodimers.^33^^,^^46^ First, we aimed to determine systemic concentratons of all five FHR proteins in serum samples of individuals with AMD and of control individuals. Until recently, the high degree of homology between members of the FH protein family and the presence of homo- and heterodimers presented a major challenge for their accurate measurement in serum. However, we used recently validated in-house-developed ELISAs to measure each protein and dimer specifically.^32^, ^33^, ^34^ Here, we measured serum concentrations of FH, FHR-1, FHR-2, FHR-3, and FHR-4, FHR-5, and the homo- and heterodimers of FHR-1 and FHR-2 in a cohort of 418 individuals, including 202 control individuals and 216 individuals with advanced AMD. Details of the study cohort are described in Table S1.

**Figure 1:**
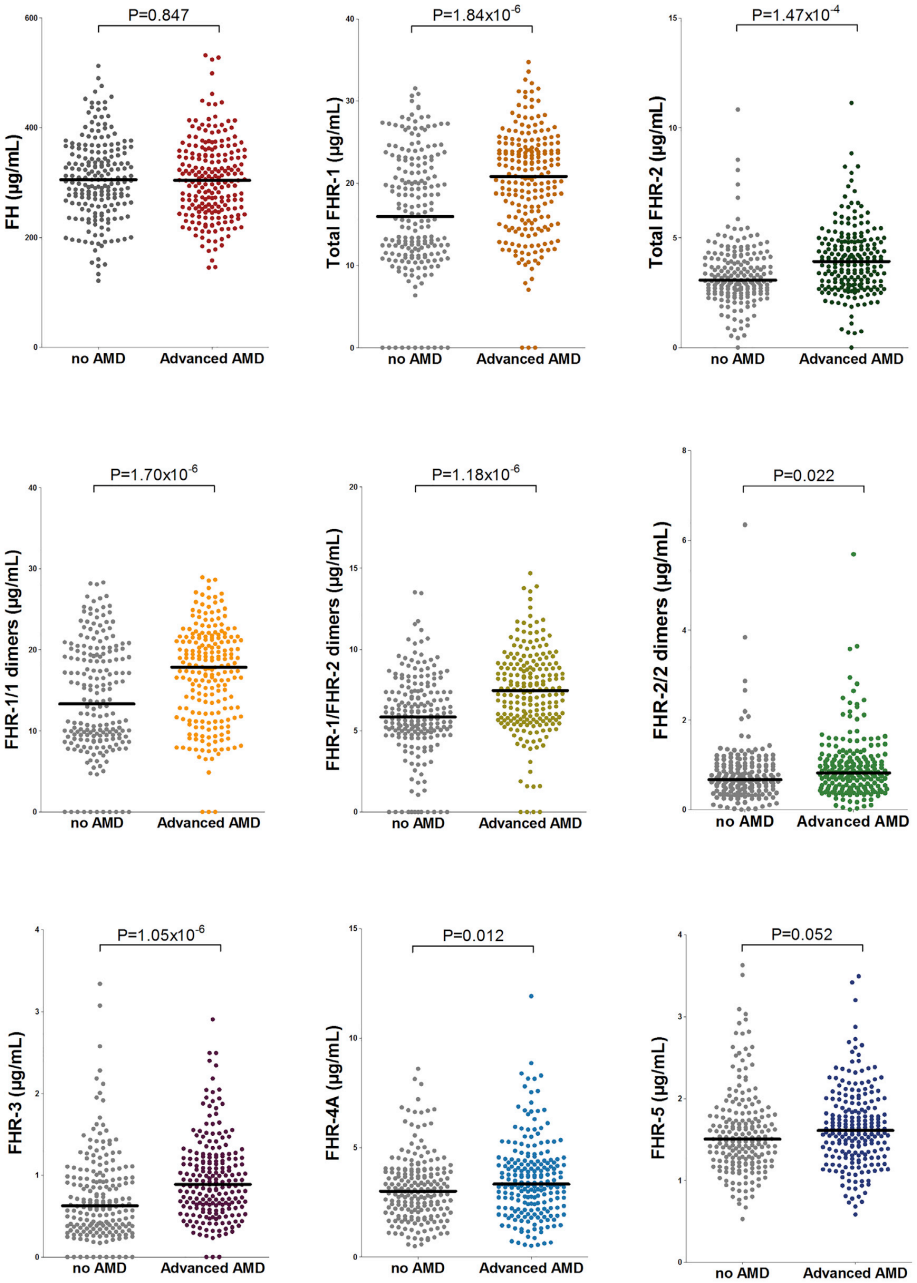
Distribution of FH, total FHR-1, total FHR-2, FHR-1/1, FHR-1/2, FHR-2/2, FHR-3, FHR-4A, and FHR-5 concentrations in individuals with advanced AMD and controls After FDR correction (see Table S3), significant differences are found for concentrations of total FHR-1, total FHR-2, FHR-1/1 dimers, FHR-1/2 dimers, FHR-2/2 dimers, FHR-3, and FHR-4A. The median value is depicted in each group.

We performed an association analysis of FHR and FH serum concentrations with advanced AMD. After correction for multiple testing, total FHR-1, total FHR-2, FHR-1/1 dimers, FHR-1/2 dimers, FHR-2/2 dimers, FHR-3, and FHR-4A concentrations were found to be associated with advanced AMD, and for all FHR proteins, an increase in systemic concentrations was associated with an increased risk for advanced AMD (Table S3, Figure 1). The strongest effect on AMD risk was observed for total FHR-1 concentrations (OR per SD increase = 2.14, 95% CI = 1.56–2.92, p_FDR_ = 5.52 × 10^−6^), followed by FHR-1/2 dimer concentrations (OR = 2.04, CI = 1.53–2.73, p_FDR_ = 5.52 × 10^−6^) and FHR-1/1 dimer concentrations (OR = 1.99, CI = 1.50–2.64, p_FDR_ = 5.52 × 10^−6^). Additional significant associations were found for FHR-3 concentrations (OR = 1.69, CI = 1.34–2.14, p_FDR_ = 2.36 × 10^−5^), total FHR-2 concentrations (OR = 1.65, CI = 1.28–2.15, p_FDR_ = 2.64 × 10^−4^), FHR-2/2 dimer concentrations (OR = 1.33, CI = 1.04–1.70, p_FDR_ = 0.03), and FHR-4A concentrations (OR = 1.33, CI = 1.06–1.65, p_FDR_ = 0.02). No significant differences were found between advanced AMD and controls for FHR-5 and FH concentrations (p_FDR_ = 0.058 and p_FDR_ = 0.847, respectively).

### AMD variants and haplotypes at the extended *CFH* locus are strongly associated with FHR-1, FHR-2, FHR-3, FHR-4A, and FHR-5 concentrations

**Figure 2:**
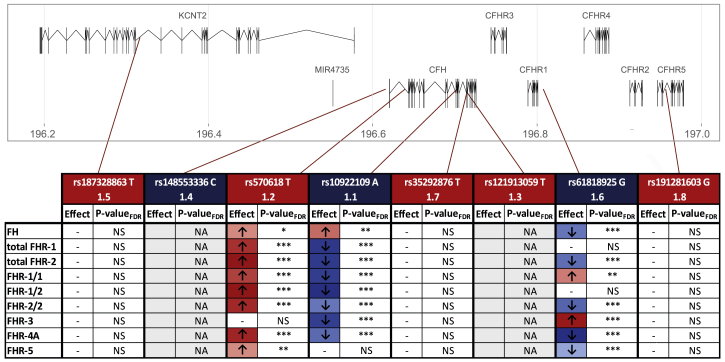
Associations of systemic FH, FHR-1, FHR-2, FHR-3, FHR-4A, and FHR-5 with AMD variants at the *CFH* locus The variants depicted are the lead variants identified in the International AMD Genomics Consortium AMD GWAS at the extended *CFH* locus in chromosome 1.^15^ Variants in blue are protective for advanced AMD and variants in red are risk-conferring in the primary analysis. The number refers to the signal identification order. If a p value is lower than 0.05, it is represented with one star (^∗^), if it is lower than 0.01, with two (^∗∗^), and if it is lower than 0.001, with three stars (^∗∗∗^). A dash (-) indicates a non-significant p value. The rare variant rs121913059 (1.3) was not present in our cohort, and the variant rs14855336 (1.4) was present in only one individual. Therefore, no analyses were performed for these two variants (gray). NS = non-significant; NA = not applicable (variant not analyzed)

Next, we set out to determine the effect of genetic variants at the *CFH* locus on FHR concentrations. The largest GWAS carried out to date on advanced AMD included 17,832 controls and 16,144 individuals with advanced AMD of European descent and identified 52 independent genome-wide-significant signals.^15^ Out of these 52 signals, eight were located at an extended *CFH* locus encompassing *CFH* and *CFHR*. The lead variants of these signals were either common in the population (rs570618, rs10922109, and rs61818925), present at a low frequency (minor-allele frequency [MAF] 1%–5%; rs187328863) or rare (MAF < 1%; rs148553336, rs35292876, rs121913059, and rs191281603). Out of the eight lead variants, one is a missense variant in *CFH* (rs121913059, FH p.Arg1210Cys), one is a synonymous variant in *CFH* (rs35292876, FH p.His878His), and the remaining variants are non-coding: intronic in *CFH* (rs570618 and rs10922109), intronic in *CFHR5* (rs191281603), intronic in *KCNT2* (rs187328863), and intergenic (rs148553336 upstream of *CFH* and rs61818925 downstream of *CFHR1* and upstream of *CFHR4*) (Figure 2). The intronic rs570618 variant is in high LD with the p.Tyr402His variant. We performed a comprehensive association analysis of these AMD variants with serum concentrations of FH and FHR.

Strong associations with FHR concentrations in serum were found for the three common variants rs10922109, rs570618, and rs61818925 (Figure 2, Table S4). The top variant of the first signal of the AMD GWAS, rs10922109 (1.1), is a protective variant that was associated with decreased serum concentrations of total FHR-1 and FHR-2, FHR-1/1 dimers, FHR-1/2 dimers, FHR-2/2 dimers, FHR-3, and FHR-4A. The strongest association was found with FHR-1/2 dimers (β = −0.749, SE = 0.064, p_FDR_ = 5.27 × 10^−26^, Table S4, Figure S2). Although to a lesser degree, rs10922109 also showed an association with increased concentrations of FH. The second-most common variant of the AMD GWAS, rs570618 (1.2, in high LD with rs1061170 [p.Tyr402His]), is an AMD-risk-conferring variant and was strongly associated with increased serum concentrations of total FHR-1 and FHR-2, FHR-1/1 dimers, FHR-1/2 dimers, FHR-2/2 dimers, FHR-4A, and to a lesser degree, FHR-5. Similar to findings for rs10922109, the strongest association was found with FHR-1/2 dimers (β = 0.587, SE = 0.064, P_FDR_ = 2.21 × 10^−17^, Table S4, Figure S2). rs570618 was associated with increased FH concentrations as well, but to a much lesser degree. The third-most common variant is rs61818925, the sixth signal of the AMD GWAS (1.6). rs61818925 was identified as protective in the single-variant analysis but was reported to be risk conferring in the locus-wide conditioned analysis.^15^ The variant rs61818925 was associated with decreased serum concentrations of FH, total FHR-2, FHR-2/2 dimers, FHR-4A, and FHR-5 and with increased serum concentrations of FHR-1/1 dimers and FHR-3. The strongest association of this variant was found with decreased serum FHR-4A concentrations (β = −0.756, SE = 0.066, p_FDR_ = 3.16 × 10^−25^, Table S4, Figure S2). The rare variant rs121913059 (FH p.Arg1210Cys) was not present in our study cohort, and the rare variant rs148553336 was only present in one individual. The other low-frequency and rare AMD variants (rs187328863, rs35292876, and rs191281603) were not associated with FH or FHR concentrations (Table S4).

The lead variants of the eight AMD signals are not entirely independent from each other nor from the *CFHR3-CFHR1* deletion (Table S5). Moreover, the alleles of these variants might exert a cumulative or synergistic effect when located on the same haplotype. Therefore, we performed a haplotype analysis of the eight AMD variants and additionally included the *CFHR3-CFHR1* deletion (by using the tag SNP rs6677604). In the analysis we included haplotypes with an overall frequency of >1%.

As previously described,^30^ when compared with the most common haplotype (H1), five haplotypes are protective for AMD: H2, H3, H4, H5, and H7, and two are associated with AMD risk: H6 and H9 (Tables 1 and 2). The protective H3 and H7 haplotypes carry the *CFHR3-CFHR1* deletion (tagged by rs6677604). Although the protective haplotypes H2, H3, H4, and H5 have a frequency >5%, H7 is only prevalent in 1.9% of the population. All five protective haplotypes were found to be associated with FHR serum concentrations, whereas no association was found for the risk haplotypes. The risk haplotype H6 differs from H1 in only one allele, namely the rare variant rs187328863, which was present in only one individual in our dataset. The risk haplotype H9 also differs from H1 in only one allele, namely the rare variant rs35292876, which was not associated with any FHR concentrations (Tables 1 and 2, Table S4).

**Table 1:** AMD haplotypes at the extended *CFH* locus are strongly associated with FHR protein concentrations: FH, total FHR-1, total FHR-2, and FHR1-1

			**FH**				**Total FHR-1**				**Total FHR-2**				**FHR1-1**			
**H**^a^	**Overall haplotype frequency**	**OR association with AMD**^b^	**B**	**SE**	**p value**	**p**_**FDR**_	**B**	**SE**	**p value**	**p**_**FDR**_	**B**	**SE**	**p value**	**p**_**FDR**_	**B**	**SE**	**p value**	**p**_**FDR**_
**H1**	**0.375**	**Reference**	**Reference**				**Reference**				**Reference**				**Reference**			
H2	0.162	0.36	−0.180	0.098	0.066	0.149	−0.096	0.073	0.187	0.299	−1.042	0.087	<0.001	<0.001	−0.023	0.074	0.756	0.851
H3	0.148	0.30	0.252	0.105	0.017	0.045	−1.247	0.078	<0.001	<0.001	−0.496	0.105	3.45 × 10^−6^	1.46 × 10^−5^	−1.273	0.081	<0.001	<0.001
H4	0.141	0.68	−0.772	0.102	2.07 × 10^−13^	1.35 × 10^−12^	0.051	0.075	0.493	0.623	−0.224	0.091	0.014	0.039	0.089	0.075	0.238	0.365
H5	0.059	0.59	−0.245	0.144	0.091	0.182	−0.212	0.104	0.043	0.103	−0.546	0.131	3.64 × 10^−5^	1.38 × 10^−4^	−0.148	0.106	0.162	0.278
H6	0.045	1.39	−0.299	0.162	0.065	0.149	−0.043	0.119	0.720	0.836	−0.329	0.144	0.023	0.057	0.000	0.119	0.997	0.997
H7	0.019	0.44	0.274	0.248	0.270	0.395	−2.389	0.284	8.88 × 10^−16^	6.39 × 10−15	−0.445	0.291	0.128	0.236	−1.980	0.361	7.47 × 10^−8^	3.84 × 10^−7^
H8	0.010	not associated	−0.621	0.370	0.094	0.183	0.214	0.190	0.261	0.392	0.098	0.313	0.755	0.851	0.157	0.221	0.476	0.612
H9	0.025	1.54	−0.192	0.204	0.346	0.488	0.121	0.151	0.422	0.573	0.066	0.183	0.719	0.836	0.118	0.152	0.436	0.581

H = haplotype; p value < 0.001 is indicated when the obtained p value was 0.

a The variants in the haplotype are as follows: rs187328863, rs148553336, rs570618, rs6677604, rs10922109, rs35292876, rs121913059, rs61818925, and rs191281603. H1 = CTTGCCCGC, H2 = CTGGACCTC, H3 = CTGAACCGC, H4 = CTGGCCCTC, H5 = CTGGCCCGC, H6 = TTTGCCCGC, H7 = CTGAACCTC, H8 = CTTGCCCTC, and H9 = CTTGCTCGC.

b Results from the International AMD Consortium dataset.^30^

**Table 2:** AMD haplotypes at the extended *CFH* locus are strongly associated with FHR protein concentrations: FHR1-2, FHR2-2, FHR-3, FHR-4A, and FHR-5

			**FHR1-2**				**FHR2-2**				**FHR-3**				**FHR-4A**				**FHR-5**			
**H**^**a**^	**Overall haplotype frequency**	**OR association with AMD**^**b**^	**B**	**SE.**	**p value**	**p**_**FDR**_	**B**	**SE.**	**p value**	**p**_**FDR**_	**B**	**SE.**	**p value**	**p**_**FDR**_	**B**	**SE**	**p value**	**p**_**FDR**_	**B**	**SE**	**P value**	**p**_**FDR**_
**H1**	**0.375**	**Reference**	**Reference**				**Reference**				**Reference**				**Reference**				**Reference**			
H2	0.162	0.36	−0.626	0.084	7.48 × 10^−13^	4.49 × 10^−12^	−0.920	0.093	<0.001	<0.001	−0.019	0.073	0.793	0.865	−1.012	0.089	<0.001	<0.001	−0.363	0.100	3.10 × 10^−4^	0.001
H3	0.148	0.30	−1.013	0.092	<0.001	<0.001	−0.047	0.112	0.676	0.811	−0.740	0.078	<0.001	<0.001	−0.140	0.096	0.146	0.263	0.143	0.108	0.187	0.299
H4	0.141	0.68	−0.125	0.087	0.153	0.269	−0.310	0.097	0.002	0.006	0.993	0.075	<0.001	<0.001	−0.533	0.093	1.83 × 10^−8^	1.01 × 10^−7^	−0.356	0.104	0.001	0.003
H5	0.059	0.59	−0.519	0.123	2.95 × 10^−5^	1.18 × 10^−4^	−0.482	0.138	0.001	0.003	0.367	0.108	0.001	0.003	0.227	0.130	0.082	0.174	−0.369	0.145	0.012	0.035
H6	0.045	1.39	−0.254	0.139	0.069	0.151	−0.358	0.155	0.022	0.057	−0.132	0.120	0.274	0.395	0.206	0.149	0.168	0.281	−0.106	0.167	0.524	0.650
H7	0.019	0.44	−1.628	0.304	1.46 × 10^−7^	7.01 × 10^−7^	−0.069	0.308	0.823	0.884	−0.907	0.182	9.44 × 10^−7^	4.25 × 10^−6^	−0.394	0.232	0.090	0.182	0.339	0.261	0.194	0.304
H8	0.010	not associated	0.164	0.275	0.550	0.671	−0.008	0.378	0.983	0.997	0.775	0.292	0.008	0.024	−0.537	0.340	0.114	0.216	−0.006	0.387	0.987	0.997
H9	0.025	1.54	0.134	0.176	0.447	0.585	−0.023	0.196	0.909	0.949	−0.043	0.151	0.776	0.860	−0.164	0.188	0.383	0.530	0.029	0.210	0.891	0.943

See legend for Table 1.

Specific protective haplotypes were more strongly associated with decreased FHR concentrations than were single variants. The protective haplotype H2, which carries a distinct allele for rs570618, rs10922109, and rs61818925 as compared to H1, was associated with strongly reduced FHR-2 (β = −1.042, SE = 0.087, p_FDR_ < 0.001), FHR-2/2 (β = −0.920, SE = 0.093, p_FDR_ < 0.001), and FHR-4A concentrations (β = −1.012, SE = 0.089, p_FDR_ < 0.001), (Figure S3). The protective haplotypes H3 and H7 carry the *CFHR3-CFHR1* deletion (tagged by rs6677604) and were the only haplotypes associated with reduced FHR-1 (H3: β = −1.247, SE = 0.078, p_FDR_ < 0.001; H7: β = −2.389, SE = 0.284, p_FDR_ = 6.39 × 10^−15^), FHR-1/1 (H3: β = −1.273, SE = 0.081, p_FDR_ < 0.001; H7: β = −1.980, SE = 0.361, p_FDR_ = 3.84 × 10^−7^), and FHR-3 concentrations (H3: β = −0.740, SE = 0.078, p_FDR_ < 0.001; H7: β = −0.907, SE = 0.182 p_FDR_ = 4.25 × 10^−6^) (Figure S3). Haplotypes H4 and H5 were associated with reduced total FHR-2, pFHR-2/2, and FHR-5 concentrations and with increased FHR-3 concentrations. Haplotype H4 was also associated with decreased FH and FHR-4A concentrations, whereas haplotype H3 showed a modest association with increased FH concentrations, and no associations of FH concentrations were observed with other haplotypes (Figure S3).

### GWASs on FH and FHR reveal strong signals at the extended *CFH* locus and a tight link to AMD risk

**Figure 3:**
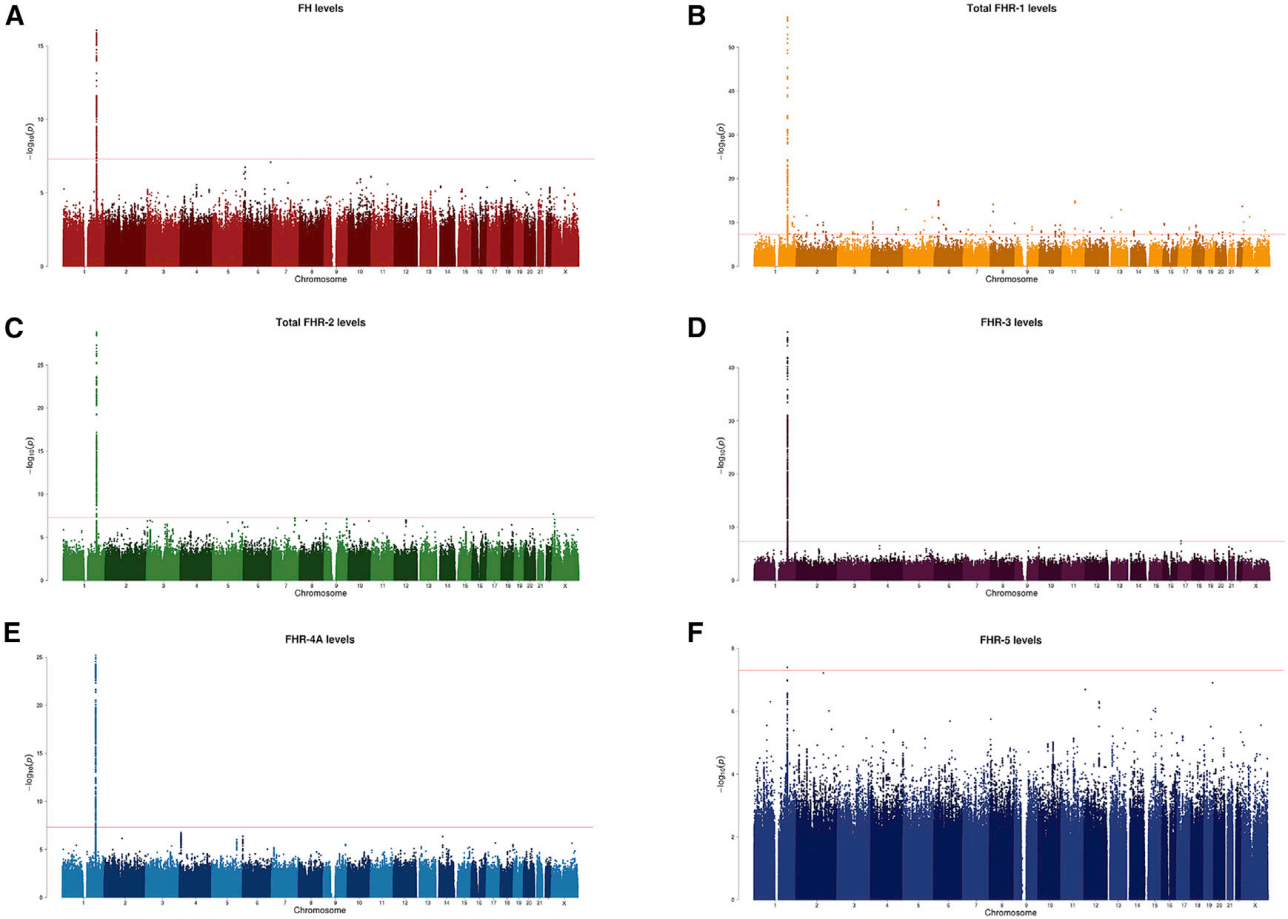
GWASs on FH and FHR concentrations identify genome-wide-significant signals in chromosome 1 at the extended *CFH* locus Manhattan plot of GWASs on FH concentrations (A), total FHR-1 concentrations (B), total FHR-2 concentrations (C), FHR-3 concentrations (D), FHR-4A concentrations (E), and FHR-5 concentrations (F) illustrate the −log (p values) of each individual variant on the basis of their chromosomal location. The red line is located at a p value of 5 × 10^−8^, which is considered the threshold for genome-wide association.

We additionally performed GWASs on FH, total FHR-1, total FHR-2, FHR-3, FHR4A, and FHR-5 concentrations to examine their genetic determinants. All proteins showed a genome-wide signal located at the extended *CFH* locus (Figure 3: Manhattan plots, Figure S4: Q-Q plots, Table 3: main results, Tables S6–S11: genome-wide-associated variants for each GWAS). After consecutive conditioning on the lead variants, the GWAS on total FHR-2 concentrations showed three additional genome-wide-significant signals at this locus, the GWAS on FHR-3 concentrations showed one additional signal, and the GWAS on FHR4-A concentrations showed two additional signals.

**Table 3:** GWAS on FH and FHR proteins concentrations identify independent genome-wide-significant signals at the extended *CFH* locus overlapping with AMD risk

**Protein**	**Signal number**	**Top variant**	**Chromosomal position**^a^	**Major/minor allele**^b^	**Location (aa change)**	**MAF**	**B**	**SE**	**p value**	**OR association with AMD**^c^	**p value association with AMD**^c^	**Linkage disequilibrium of R^2^ ≥ 0.3 with index AMD GWAS signals**
FH	FH.1	rs70620^d^	1:196704997	G/A	intronic *CFH*	0.170	−0.754	0.087	8.73 × 10−17	0.935	0.003	
total FHR-1	FHR-1.1	rs148235292	1:196814850	T/A	intergenic: downstream of *CFHR1*, upstream of *CFHR4*	0.167	−1.376	0.073	1.39 × 10^−57^	0.410	1.23 × 10^−284^	
total FHR-2	FHR-2.1	rs3790414	1:196920299	T/A	Intronic *CFHR2*	0.177	−0.979	0.080	1.59 × 10^−29^	0.537	3.16 × 10^−187^	R^2^ = 0.346 with 1.1, R^2^= 0.358 with 1.6
	FHR-2.2	rs79351096	1:196918741	G/A	exonic *CFHR2* (FHR-2 p.Cys72Tyr)	0.012	−2.268	0.241	5.07 × 10^−19^	0.634	1.01 × 10^−13^	R^2^ = 0.421 with 1.4
	FHR-2.3	rs41310132	1:196928188	A/G	exonic *CFHR2* (FHR-2 p.Tyr264Cys)	0.009	−1.833	0.279	1.65 × 10^−10^	0.408	1.54 × 10^−28^	
	FHR-2.4	rs114645367	1:196716998	G/A	intergenic downstream of *CFH*	0.012	−1.507	0.228	1.32 × 10^−10^	1.500	1.14 × 10^−7^	
FHR-3	FHR-3.1	rs529541 d	1:196719716	A/G	intergenic downstream of *CFH*	0.178	1.161	0.070	1.92 × 10^−47^	0.949	0.018	
	FHR-3.2	rs138396963	1:196724984	AC/A	intergenic downstream of *CFH*, upstream of *CFHR3*	0.343	−0.772	0.066	1.01 × 10^−27^	0.578	1.82 × 10^−202^	R^2^ = 0.308 with 1.2
FHR-4A	FHR-4A.1	rs56994749^e^	1:196813692	T/A	intergenic downstream of *CFHR1*, upstream of *CFHR4*	0.329	0.770	0.068	6.11 × 10^−26^	1.577	1.12 × 10^−135^	R^2^ = 0.763 with 1.6
	FHR-4A.2	rs10494745	1:196887457	G/A	exonic *CFHR4* (FHR-4 p.Gly552Glu)	0.099	−1.302	0.089	6.35 × 10^−39^	1.763	2.24 × 10^−121^	
	FHR-4A.3	rs10922108	1:196701473	A/T	intronic *CFH*	0.335	−0.449	0.053	2.78 × 10^−16^	0.382	1.48 × 10^−612^	R^2^ = 1 with 1.1, R^2^ = 0.411 with 1.2
FHR-5	FHR5.1	rs6695321^e^	1:196675861	A/G	intronic *CFH*	0.337	−0.398	0.071	4.03 × 10^−8^	0.616	2.24 × 10^−170^	R^2^ = 0.395 with 1.2, R 2= 0.630 with 1.6

^f^Linkage disequilibrium with index variants from the eight independent signals of the GWAS on AMD from the IAMDGC,^15^ also described in Figure 2. The matrix of pairwise linkage disequilibrium statistics was generated with “LDlink” (accessed on February 2021) and is displayed in Table S12. All European reference populations from the reference haplotypes from phase 3 (version 5) of the 1000 Genomes Project were used for the linkage-disequilibrium calculations.

a Chromosomal position is based on NCBI RefSeq hg19,

b Effect

c Extracted from the single-variant unconditioned results of the GWAS on AMD from the IAMDGC.^15^

d R^2^ for these two variants is 0.987 (“LDlink,” accessed on May 2020 and including all European reference populations from the reference haplotypes from phase 3 (Version 5) of the 1000 Genomes Project),

e R^2^ for these two variants is 0.756 (“LDlink,” accessed on May 2020 and including all European reference populations from the reference haplotypes from Phase 3 (Version 5) of the 1000 Genomes Project)The model was adjusted for age, sex, measuring batch, sampling cohort, and AMD status, as well as ten ancestry principal components. The resulting betas reflect the allele effect on log-transformed +1 and standardized protein concentrations. Second, third, and fourth signals were identified after adjustment for the lead variant(s) of the previously identified signal.

The top variant in the total FHR-1 GWAS, rs148235292, was associated with decreased FHR-1 concentrations (B = −1.376, SE = 0.073, p = 1.39 × 10^−57^), and it is also a protective variant for AMD at a genome-wide-significant level (OR = 0.41, p = 1.23 × 10^−284^). The same scenario was found for the first signal of the total FHR-2 GWAS (FHR-2.1), rs3790414, which was associated with decreased total FHR-2 concentrations (B = −0.979, SE = 0.080, p = 1.59 × 10^−29^), and it is also associated with a protective effect for AMD at a genome-wide significant level (OR = 0.54, p = 3.16 × 10^−187^). The top variant of the first signal of the FHR-4A GWAS (FHR-4A.1), rs56994749, was associated with increased FHR-4A levels (B = 0.770, SE = 0.068, p = 6.11 × 10^−26^), and with a higher risk for AMD at a genome-wide significant level (OR = 1.58, p = 1.12 × 10^−135^). The top variant for the FHR-5 GWAS, rs6695321, was associated with decreased FHR-5 concentrations (B = −0.398, SE = 0.071, p = 4.03 × 10^−8^), and it is protective for AMD at a genome-wide-significant level (OR = 0.62, p = 2.24 × 10^−170^; Table 3). Notably, the r^2^ between rs56994749 and rs6695321 (FHR-4A.1 and FHR-5.1) is 0.76, whereas the other above-mentioned lead variants have an r^2^<0.5, (Table 3 and Table S12).

The top variant of the only signal in the FH GWAS, rs70620, was associated with decreased FH concentrations and with a lower risk for AMD, but with a much lower degree of significance (OR = 0.94, p = 0.003). The top variant of the first signal of the FHR-3 GWAS (FHR-3.1), rs529541, was associated with increased FHR-3 concentrations but with a protective effect for AMD at a p value just below the 0.05 threshold (OR = 0.95, p = 0.018). These two variants, rs70620 and rs529541 (FH.1 and FHR-3.1), have an r^2^ of 0.98 (Table S12). The second signal of the FHR-3 GWAS, rs138396963 (FHR-3.2), was associated with decreased FHR-3 concntrations (B = −0.772, SE = 0.066, p = 1.01 × 10^−27^) and with a protective effect for AMD at a genome-wide-significant level (OR = 0.58, p = 1.82 × 10^−202^; Table 3).

The total FHR-2 GWAS revealed four genome-wide-significant signals, of which the FHR-2.2, FHR-2.3, and FHR-2.4 signals were low-frequency variants. The top associated variants of the FHR-2.2 and FHR-2.3 signals, rs79351096 and rs41310132, are missense variants in FHR-2, leading to p.Cys72Tyr and p.Tyr264Cys. Both were associated with decreased concentrations of FHR-2 and a protective effect for AMD at genome-wide-significant level. The top variant of the FHR-2.4 signal, rs114645367, was associated with decreased FHR-2 concentrations but a higher risk for AMD. The FHR-4A GWAS revealed three genome-wide-significant signals. The top variant of the FHR-4A.2 signal, rs10494745, is a missense *CFHR4* variant that was associated with decreased FHR-4A concentrations but with an increased risk for AMD at a genome-wide-significant level. Finally, the top variant of the FHR-4A.3 signal, rs10922108, was associated with decreased FHR-4A concentrations and with a protective effect for AMD at a genome-wide-significant level (Table 3). Interestingly, rs148235292 (FHR-1.1) is in LD (r^2^ = 0.94) with rs6677604, the tag variant for the *CFHR3-CFHR1* deletion, and rs10922108 (FHR-4A.3) is in LD (r^2^ = 1) with the AMD GWAS 1.1 top variant rs10922109 (Table 3, Table S12). Several index variants of these FHR GWAS signals are in some degree of LD with known index variants of AMD GWAS signals (Table 3 and Table S12).

### Low-frequency and rare variants in *CFHR2* and *CFHR5* are associated with advanced AMD

The largest GWAS on advanced AMD was performed on an exome-chip platform that included not only common variants but also low-frequency (MAF = 1%–5%) and rare (MAF < 1%) variants. The evaluation of the effect of rare protein-altering variants in *CFH* revealed a burden associated with advanced AMD independently of the eight signals identified in the GWAS.^15^ Nonetheless, the effect of low-frequency variants on AMD has not been evaluated yet. We identified p.Cys72Tyr and p.Tyr264Cys (low-frequency variants) as lead variants of the total FHR-2 GWAS. We identified an association of systemic FHR-2 concentrations with advanced AMD and, therefore, reasoned that low-frequency and rare variants in *CFHR2* and other *CFHR* might be associated with AMD risk. Because such protein-altering variants most likely impact protein function, they can help pinpoint causal genes for AMD. Using the IAMDGC dataset, we performed gene-based tests for protein-altering low-frequency and rare variants in *CFHR1*, *CFHR2*, *CFHR3*, *CFHR4*, and *CFHR5* (and *CFH* for consistency). A total of 17,596 controls and 15,894 individuals who had advanced AMD in the IAMDGC dataset and were of European descent were included in this analysis.^15^ We also performed the association tests after adjusting for the eight previously reported AMD signals at the extended *CFH* locus^15^ in order to rule out the possibility that the associations were driven by previously described signals.

Associations of protein-altering low-frequency and rare variants with advanced AMD in the locus-wide conditioned analysis were found for the *CFH*, *CFHR2*, and *CFHR5* (SKAT-O p values of 7.52 × 10^−6^, 5.03 × 10^−3^, and 2.81 × 10^−6^, respectively; Table 4). Sensitivity analysis with adjustment for seven additional ancestry principal components confirmed lack of confounding due to population stratification (Table S13). The gene-based test for *CFH* included 55 variants with 13 singletons, for *CFHR2* included 11 variants with three singletons, and for *CFHR5* included 30 variants with 13 singletons (Table 4 and Table S14; details of all the variants included in each test are provided).^47^

**Table 4:** Gene-based tests identify low-frequency and rare variants in *CFH*, *CFHR2*, and *CFHR5* associated with age-related macular degeneration

**Gene**	**Chromosomal location**^a^	**Number of variants included**	**Number of singletons included**	**Number of individuals carrying low-frequency and rare variants**	**AC in AMD**	**AC in controls**	**Unconditioned p value**^b^	**p value conditioned on common variants**^c^	**Locus-wide conditioned p value**^d^	**SKAT-O ρ**^e^
*CFH*	1:196621254–196716353	55	13	2,077	922	1,364	8.63 × 10−60	4.50 × 10−13	7.52 × 10−6	0
*CFHR1*	1:196794703–196797262	4	2	11	6	5	0.669	0.557	0.601	1
*CFHR2*	1:196918615–196928188	11	3	3,177	1,262	2,026	2.52 × 10−53	5.87 × 10−10	0.005	0.3
*CFHR3*	1:196748349–196748468	4	2	31	15	16	0.179	0.545	0.578	0
*CFHR4*	1:196871563–196887530	20	1	555	191	378	1.47 × 10−11	0.363	0.408	0
*CFHR5*	1:196952046–196977807	30	10	2,771	1,247	1,708	4.78 × 10−29	9.57 × 10−18	2.81 × 10−6	0

AC = allele count. SKAT-O gene-based tests were carried out for comparing protein-altering variants with a minor-allele frequency < 0.05 in 17,596 controls and 15,894 individuals with advanced AMD (a total of 33,488 individuals). Bonferroni correction for multiple testing was applied, and p values > 0.008 were considered statistically significant. Details about the variants included in the test are displayed in Table S14.

a Chromosomal position according to the NCBI RefSeq hg19 human genome reference assembly.

b SKAT-O tests were adjusted for the first two ancestry principal components and source of DNA (whole-blood or whole-genome amplified DNA).

c SKAT-O test was additionally adjusted for the common index variants in the locus that have been previously reported to be associated with AMD (rs570618, rs109221099 and rs61818925^15^).

d SKAT-O test was additionally adjusted for all the index variants in the locus that have been previously reported to be associated with AMD (rs187328863, rs148553336, rs570618, rs10922109, rs35292876, rs121913059, rs61818925, and rs191281603^15^).

e As computed in the locus-wide conditioned analysis.

**Figure 4:**
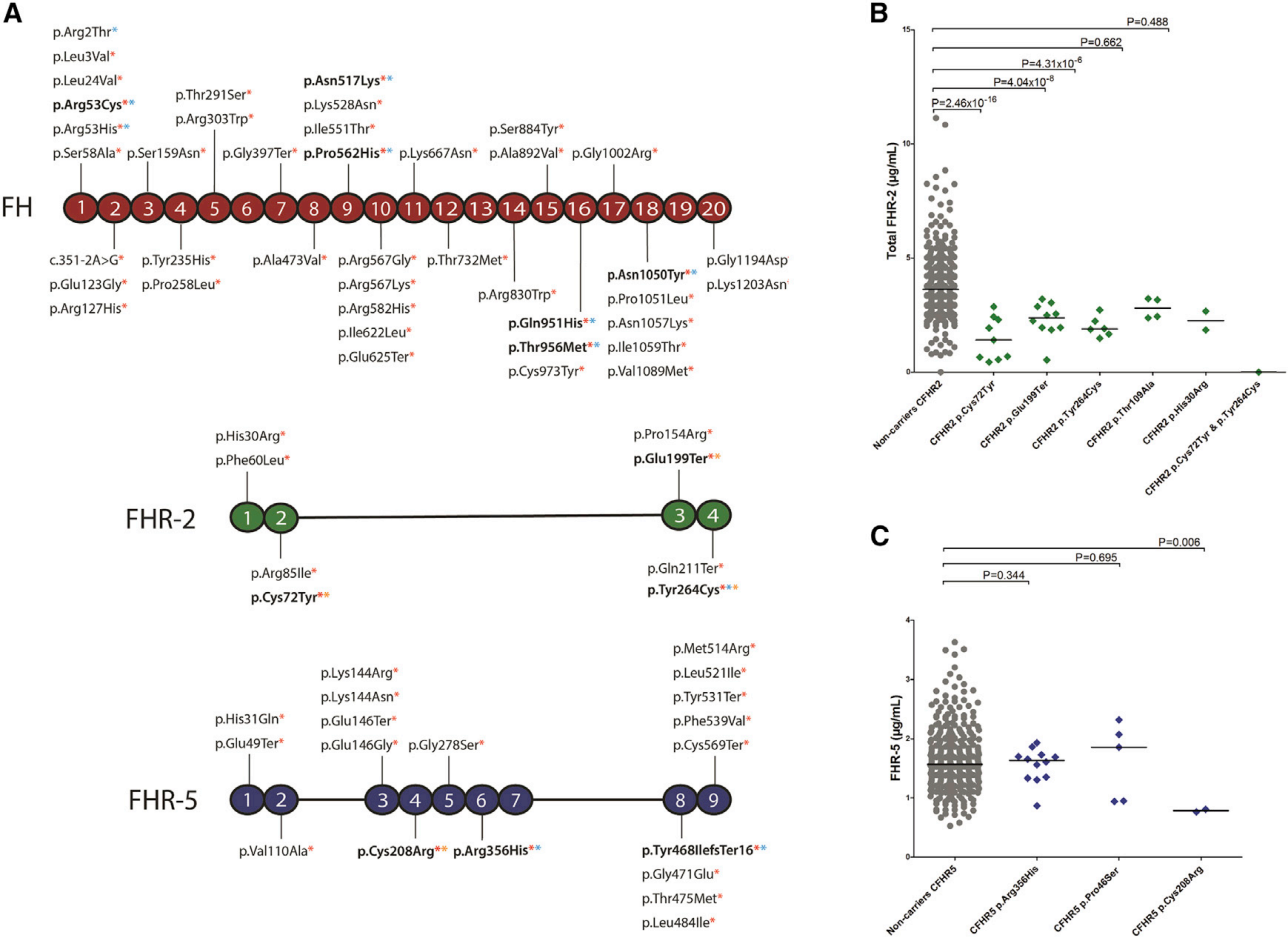
Low-frequency and rare variants in CFHR genes and age-related macular degeneration (A) Schematic representation of FH, FHR-2, and FHR-5, including variants analyzed in the gene-based test with a PHRED-CADD score > 10 (red asterisk), those with an AMD association independent of previously reported GWAS signals (p value < 0.05; blue asterisk), and those with an identified effect on protein concentrations (yellow asterisk). Variants that meet two or more criteria are highlighted in bold. (B) Distribution of total FHR-2 serum concentrations in individuals carrying low-frequency *CFHR2* variants included in the gene-based test (C) Distribution of FHR-5 serum concentrations in individuals carrying low-frequency *CFHR5* variants included in the gene-based test. Shown p values are unadjusted (see Table 4 for FDR-adjusted p values). The median value is depicted in each group.

We additionally performed single-variant association analysis for the variants included in the gene-based tests of *CFH*, *CFHR2* and *CFHR5* in order to assess whether the direction of the effect of these variants could be determined. This analysis was also adjusted for the eight previously reported AMD signals.^15^ A total of 11 variants had p < 0.05; these included two variants in FHR-5 (p.Arg356His [OR = 0.75, p = 7.88 × 10^−7^] and p.Tyr468IlefsTer16 [OR = 7.19, p = 0.020]) and one variant in FHR-2 (p.Tyr264Cys [OR = 0.75, p = 6.60 × 10^−6^] (Table S14, Figure 4A).

Several tools can help with predicting the deleteriousness of genetic variants. The tool “combined annotation-dependent depletion” (CADD) integrates multiple annotations into one metric.^48^ Variants with PHRED-scaled CADD scores higher than 10 are predicted to be the 10% most deleterious substitutions in the human genome. We performed an additional sensitivity analysis including only variants with a PHRED-scaled CADD score > 10. We found stronger associations for all three genes (*CFH, CFHR2*, and *CFHR5*) when we included only variants with a PHRED-scaled CADD score > 10 (SKAT-O p = 2.77 × 10^−6^, 3.26 × 10^−3^ and 8.62 × 10^−7^, respectively; Table S15, Figure 4A).

### Low-frequency variants in *CFHR2* and *CFHR5* are associated with decreased systemic concentrations of FHR-2 and FHR-5

Finally, we evaluated whether carriers of variants included in the gene-based tests of *CFH*, *CFHR2*, and *CFHR5* have altered protein concentrations in their serum. A total of 12 variants were present in more than one individual within our cohort; therefore, serum measurements of FH, FHR-2, and FHR-5 were available for these individuals (Table S16). Low-frequency and rare variants in *CFHR2* were associated with decreased serum FHR-2 concentrations in individuals with the p.Cys72Tyr (β = −2.334, SE = 0.272, p_FDR_ = 2.95 × 10^−15^), p.Glu199Ter (β = −1.491, SE = 0.266, p_FDR_ = 2.42 × 10^−7^), and p.Tyr264Cys (β = −1.524, SE = 0.327, p_FDR_ = 1.72 × 10^−5^) variants (Figures 4A and 4B). The p.Cys208Arg variant in *CFHR5* was associated with decreased FHR-5 concentrations (β = −1.869, SE = 0.679, p_FDR_ = 0.018; Table S16, Figures 4A and 4C). Carriers of rare variants in *CFH* did not have altered FH concentrations (Table S16).

### FHR-2 and FHR-5 localize in the interface between the retinal pigment epithelium and the choroid

**Figure 5:**
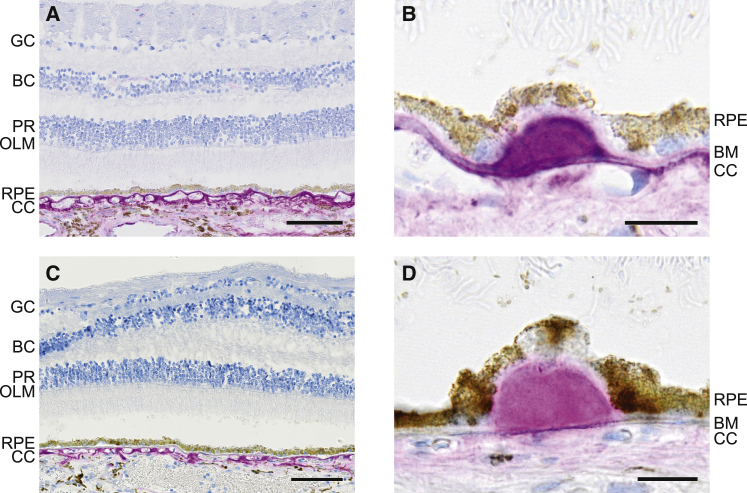
Immunohistochemistry of human retinas shows localization of FHR-2 and FHR-5 in the choriocapillaris and in drusen GC = ganglion cells; BC = bipolar cells; PR = photoreceptors; OLM = outer limiting membrane; RPE = retinal pigment epithelium; CC = choriocapillaris, and BM = Bruch’s membrane. To circumvent the difficulties originating from the intense autofluorescence of retinal/choroidal structures (e.g., lipofuscin granules, drusen, and capillary walls), we used a non-fluorescent immunolabeling method to detect FHR-2 and FHR-5 in a non-AMD eye. The distributions of the two proteins are indistinguishable and confined to the choroid (A and C, for FHR-2 and FHR-5, respectively; the scale bar represents 100 μm). The highest signal intensities were detected in the connective tissue surrounding the choriocapillaris. Similarly, strong signal can be detected in the Bruch’s membrane and in drusen (B and D, for FHR-2 and FHR-5, respectively; the scale bar represents 20 μm). Sections were incubated with the primary antibodies anti-FHR-2.1 from Sanquin for FHR-2 and anti-FHR-5.4 from Sanquin for FHR-5. The antibody anti-FHR-2.1 was generated against FHR-2 but also recognizes FHR-1. Anti-FHR-5.4 is monospecific for FHR-5; therefore, staining with a monoclonal antibody found to recognize FHR-2 exclusively (R&D Systems, MAB5484) was also performed (Figure S5).

In this study, we describe an association of FHR-2 and FHR-5 with AMD. We additionally assessed whether they can also be detected at sites where the pathological changes manifest in the eye. We investigated the localization of these proteins in human ocular tissue sections by using anti-FHR-2 and anti-FHR-5 antibodies.^33^ Strikingly, both FHR-2 and FHR-5 were detected in the intercapillary septa and the extracellular matrix surrounding the choriocapillaris (Figure 5 and Figure S5). A signal in the deeper layers of the choroid was also present, but it was fainter than in the intercapillary septa. The signal intensities varied significantly within the same layer but in different areas of a single section, where regions with strong deposit accumulation exhibited the strongest staining. Sub-RPE drusen showed strong immunolabeling for both FHR-2 and FHR-5, whereas no immunostaining was detected in the neuroretina. Importantly, no intracellular staining was detected for either FHR-2 or FHR-5 in the choroid-RPE interface, indicating an extraocular (hepatic) origin (Figure 5 and Figure S5).

## Discussion

In the present study, we measured FH and FHR proteins in serum of individuals with advanced AMD and controls by using ELISAs that allowed for specific quantification, despite a high level of homology between these proteins. We identified elevated systemic concentrations of total FHR-1, total FHR-2, FHR-1/1 dimers, FHR-1/2 dimers, FHR-2/2 dimers, FHR-3, and FHR-4A in individuals with AMD, and we found associations of all FHR concentrations with common AMD genetic variants and haplotypes at the extended *CFH* locus. In addition, we identified low-frequency and rare *CFHR2* and *CFHR5* variants associated with AMD independently of the previously reported GWAS signals. Low-frequency variants in *CFHR2* and *CFHR5* also led to decreased or absent FHR-2 and FHR-5 concentrations. Finally, we observed that FHR-2 and FHR-5 localize in the choriocapillaris, specifically in the intercapillary septa, as well as in drusen, the hallmark lesions of AMD.

Our study reports elevated concentrations of total FHR-1, total FHR-2, FHR-1/1 dimers, FHR-1/2 dimers, FHR-2/2 dimers, FHR-3, and FHR-4A in AMD. The association between increased FHR-1 and FHR-3 concentrations and advanced AMD is in line with the previously reported protective effect of the *CFHR3-CFHR1* deletion.^22^, ^23^, ^24^, ^25^, ^26^, ^27^, ^28^, ^29^ Our findings suggest that the deletion exerts its protective effect on AMD by decreased or absent FHR-1 and FHR-3 in the carriers. However, this finding contrasts with a previous study that described decreased FHR-1 concentrations in individuals with AMD.^49^ The authors claimed that the effect of the *CFHR3-CFHR1* deletion could therefore not be mediated by reduced concentrations of FHR-1 and would be mediated by increased FH concentrations instead. However, the ELISA used in that study showed protein concentrations of FHR-1 in *CFHR1*-deficient donors, suggesting that the assay is not specific for FHR-1. A recent study reported elevated FHR-4A concentrations in advanced AMD in a meta-analysis of two independent cohorts,^30^ measured with a different anti-FHR-4A antibody, which is confirmed in our current study. FHR-5 was the only FHR that was not associated with AMD in our analysis; however, the p value was close to significance (p = 0.052). Therefore, an extended analysis with a larger sample size might confirm elevated FHR-5 concentrations in AMD.

Importantly, we did not observe any differences in systemic FH concentrations between individuals with advanced AMD and controls. Previous studies have reported conflicting results on this regard.^30^^,^^49^, ^50^, ^51^, ^52^, ^53^, ^54^ Potential explanations might be differences in size and ethnicity of the cohorts; cross-reactivity of the assays with FHL-1,^30^^,^^49^^,^^50^ FHR-1,^51^ or both FHL-1 and FHR-1;^50^^,^^52^^,^^55^ or unclear cross-reactivity.^53^^,^^54^^,^^56^ In the current study, we used an antibody that targets CCP16/17 in FH but does not detect any other member of the FH protein family, allowing us to specifically measure systemic FH only.^35^ We conclude that systemic FHR concentrations and not FH concentrations are associated with advanced AMD.

We also find that, out of the eight AMD signals reported in the large AMD IAMDGC GWAS at the extended *CFH* locus,^15^ all three common variants were strongly associated with FHR protein concentrations. An association of these variants with total FHR-4A has been recently described,^30^ but in the current study we demonstrate that this association extends to all five FHR proteins.

We report that the AMD GWAS top variant 1.1, rs10922109, associates with altered FHR-1, FHR-2, FHR-3, and FHR-4A concentrations. This variant has recently been described to be an eQTL for *CFHR1*, *CFHR3*, and *CFHR4* in liver.^57^ The top variant of the 1.2 AMD GWAS signal, rs570618, is in tight LD with rs1061170 (p.Tyr402His) and associates with altered FHR-1, FHR-2, FHR-4A, and FHR-5 concentrations. Our findings suggest that the strong effect of the rs1061170 (p.Tyr402His) genotype on AMD risk might be due to the altered FHR concentrations associated with this variant, which has also recently been reported to be an eQTL for *CFHR1* in liver.^57^ In a recent publication, it was reported that the p.Tyr402His variant modulates FH recruitment and its ability to regulate complement on malondialdehyde epitopes and that FHR-1 can compete with FH for binding to malondialdehyde *in vitro*.^58^ The association that the current study identified for elevated FHR-1 concentrations with rs570618 and thus p.Tyr402His suggests that elevated FHR-1 concentrations contribute to the altered ability of FH to bind to malondialdehyde in individuals with the rs1061170 (p.Tyr402His) genotype. Notably, in that study, a cluster of variants located in *CFHR2*, *CFHR4*, and *CFHR5* showed an effect on FH binding to MDA independently of the rs1061170 (p.Tyr402His) genotype, suggesting that other FHR proteins might also exert such an effect.^58^ The top variant of the 1.6 AMD GWAS signal, rs61818925, is associated with FHR-2, FHR-3, FHR-4A, and FHR-5 concentrations. This variant has been reported to be an eQTL for *CFHR3* and *CFHR5* in liver.^57^ It was also found to be an eQTL for *CFHR1*,^57^ and the uncorrected p value of the association with FHR-1 concentrations in our study is 0.03, suggesting that this variant might also affect FHR-1 concentrations. Although all three variants associate with concentrations of several FHR proteins, the underlying mechanisms of these associations remain to be elucidated in future studies.

Additionally, we find stronger effects of haplotypes than of individual genotypes on FHR concentrations. AMD-protective haplotypes H2, H3, H4 H5, and H7 are generally associated with reduced FHR concentrations. The strongest effects were identified for the H2 protective haplotype, which associated with reduced FHR-2 and FHR-4A concentrations. Our haplotype analysis indicates that assessing haplotypes is more informative when assessing AMD risk, and especially the effects on FHR protein concentrations, at this locus.

GWASs on FH and all FHR protein concentrations identified genome-wide associations at the extended *CFH* locus, and we observe that FHR concentrations have, in most cases, different genetic determinants. GWASs on FHR-1, FHR-2, FHR-3, and FHR-5, which have not been carried out to date with specific antibodies, revealed one (FHR-1 and FHR-5), four (FHR-2), and two (FHR-3) independent signals at the extended *CFH* locus. A recent study that has analyzed associations with FH at a genome-wide level reported the strongest association for rs10784193 on chromosome 12.^58^ In our GWAS this variant had a p value of 0.300 and thus was not associated with FH concentrations. The index variant in our FH GWAS, rs70620, was associated with decreased FH concentrations but was only nominally associated with a lower risk for AMD, and we found a relatively small effect size and a p value that did not reach genome-wide significance in the AMD GWAS (OR = 0.94, p = 0.003). After conditioning for rs70620, we did not find associations for any other variants with FH concentrations at a genome-wide significant level, confirming that there is not an evident association between systemic FH concentrations and AMD. A GWAS on FHR-4A had been previously performed in a study using a different antibody and had shown comparable results.^30^ Several index variants of these FHR GWAS signals were also strongly associated with AMD and in LD with reported index variants of AMD GWAS signals (Table 3 and Table S12). Therefore, FHR-associated variants could be underlying the known AMD associations at the extended *CFH* locus. Genetic variations in CFHR genes have also been reported to be involved in other complement diseases.^59^ Consequently, the signals that we have identified could be further explored in future studies in those contexts.

Protein-altering variants most likely impact protein function and can help pinpoint causal genes. We analyzed the effect of low-frequency and rare variants in CFHR genes on AMD, independently of all previously reported signals, in 17,596 controls and 15,894 individuals who had advanced AMD and were included in the IAMDGC GWAS.^15^ Low-frequency and rare functional variants in *CFHR2* and *CFHR5* were associated with advanced AMD, which directly implicates these genes in the disease and also shows that additional genetic associations are present at this locus.

Of the variants included in the gene-based test, 68 variants had a PHRED-CADD score >10, demonstrated an individual association with AMD (p < 0.05) independently of previously reported GWAS signals,^15^ or had an effect on protein concentrations (p < 0.05), and 11 variants met two or more of these criteria (Figure 4A). In FH, p.Arg53Cys, p.Asn517Lys, p.Pro562His, p.Gln950His, p.Thr956Met, and p.Asn1050Tyr had a PHRED-CADD score >10 and showed an association with AMD. These variants had been previously described in AMD, but we also report p < 0.05 for p.Asn517Lys, p.Pro562His, and p.Thr956Met.^60^

In FHR-2, p.Cys72Tyr, p.Glu199Ter, and p.Tyr264Cys had a PHRED-CADD score >10 and a lowering effect on total FHR-2 protein concentrations. p.Cys72Tyr and p.Glu199Ter had been previously reported to result in a complete absence of FHR-2, in the case of the p.Cys72Tyr variant because of a disulfide bridge that cannot be formed.^33^ The p.Tyr264Cys variant introduces a cysteine residue and is likely to affect folding of FHR-2. The p.Tyr264Cys variant additionally shows a protective effect on AMD independently of previously reported signals (OR = 0.75, p = 6.60 × 10^−4^). The p.Tyr264Cys variant in *CFHR2* had been recently highlighted in the context of AMD.^29^ In that study, the extended *CFH* locus was sequenced with molecular inversion probes. Single-variant analysis was performed and was followed by a meta-analysis that included 1,574 AMD cases and 855 controls from three cohorts.^29^ The p.Tyr264Cys variant was found to be associated with AMD in an analysis adjusted for age, sex, and the p.Tyr402His variant (OR = 0.37, p = 1.45 × 10^−3^). Interestingly, p.Cys72Tyr is in some degree of LD (r^2^ = 0.421, Table S12) with rs148553336, the 1.4 signal of the AMD GWAS. The rs148553336 variant is a low-frequency, non-coding variant located upstream of *CFH*; therefore, the protective effect of the 1.4 signal in the AMD GWAS could be partially reflecting a protective effect of p.Cys72Tyr on AMD.

Finally, in FHR-5, p.Cys208Arg had a PHRED-CADD score >10 and a lowering effect on total FHR-5 protein concentrations. The p.Cys208Arg variant most likely interferes with the formation of a disulfide bridge, leading to retention of the aberrantly folded FHR-5 protein in the cell. The p.Arg356His and p.Tyr468IlefsTer16 variants had a PHRED-CADD score >10 and showed an association with AMD (OR = 0.75, p = 7.88 × 10^−7^ and OR = 7.19, p = 0.02, respectively). A protective effect would have been expected for the frameshift variant and therefore requires further research. Gene-based tests often arbitrarily analyze rare variants with a MAF < 1%. The FHR-2 variants p.Cys72Tyr, p.Glu199Ter, and p.Tyr264Cys are all low-frequency, which highlights the importance of analyzing variants with a MAF < 5%. Low-frequency variants may therefore be also analyzed in other AMD loci or throughout the genome, as they might play a relevant role in AMD.

The effects that we report are at a systemic level, and the local effects at the AMD disease site remain to be elucidated. We describe localization of FHR-2 and FHR-5 to the intercapillary septa of the choriocapillaris and in drusen. The lack of the immunopositive cells in the choroid and RPE suggests that these FHR proteins derive from the systemic circulation entering the choroidal stroma through the fenestration of the choriocapillaris and diffusion through the Bruch’s membrane. This has been previously described as well for FHR-4A.^30^ Alterations in systemic FHR proteins abundance may therefore impact pathogenesis locally at the AMD disease site.

Rare protein-altering variants impairing the function of FH or FHL-1^60^^,^^61^ and common variants at the extended *CFH* locus that increase FHR concentrations could shift the delicate balance between FH and FHR in the choriocapillaris and Bruch’s membrane and thus increase the risk for AMD. On the other hand, low-frequency variants leading to low or absent concentrations of FHR-2 and FHR-5 are protective for AMD. Our findings highlight the FHR proteins as potential drug targets that could be inhibited for the treatment or prevention of disease progression in AMD. Selection of individuals with AMD for FHR-inhibiting therapy would need to involve consideration of both common haplotypes in the extended *CFH* locus, as well as low-frequency and rare variants in *CFHR2* and *CFHR5*.

In conclusion, our results deepen the understanding of the effects of genetic variation at the extended *CFH* locus and pinpoint a relevant role of FHR proteins in AMD; these results are also supported by a recent study by Unwin et al.^62^ We describe the involvement of FHR-2 and FHR-5 in AMD and identify low-frequency *CFHR2* and *CFHR5* variants with a protective effect on AMD. Our study could set a precedent for the analysis of other GWAS loci and pinpoints FHR proteins as potential targets for developing new AMD treatments where individuals with AMD could be selected on the basis of their genetic profile.

## Declaration of interests

A.E., J.L.M., C.S., E.N., E.K., and S.F. are employees of F. Hoffmann-La Roche. S.C. is an inventor on patent applications that describe the use of complement inhibitors for therapeutic purposes and is a co-founder and director of Complement Therapeutics. T.W.K. is coinventor on a patent (PCT/NL2015/050584) describing the potentiation of FH with mAbs and therapeutic uses thereof. A.I.d.H. is a consultant for Ionis Pharmaceuticals, Gyroscope Therapeutics, Gemini Therapeutics, and F. Hoffmann-La Roche.

## Data and code availability

GWAS results will be accessible at the GWAS catalog repository (https://www.ebi.ac.uk/gwas/).

Serum FH concentrations GWAS GCST90019041

Serum FHR-1 concentrations GWAS GCST90019042

Serum FHR-2 concentrations GWAS GCST90019043

Serum FHR-3 concentrations GWAS GCST90019044

Serum FHR-4A concentrations GWAS GCST90019045

Serum FHR-5 concentrations GWAS GCST90019046

## Web resources

CADD, https://cadd.gs.washington.edu
EPACTS, http://genome.sph.umich.edu/wiki/EPACTS
EUCLIDS, www.euclids-project.eu
LDlink, https://ldlink.nci.nih.gov

## Supplemental information

Document S2. Tables S2, S4–S12, and S14

Document S3. Article plus supplemental information
