# Supplementary material for: Common haplotypes at the CFH locus and low-frequency variants in CFHR2 and CFHR5 associate with systemic FHR concentrations and age-related macular degeneration
Source: Am J Hum Genet. 2021 Jul 13;108(8):1367–84. doi: 10.1016/j.ajhg.2021.06.002 (PMC8387287; doi:10.1016/j.ajhg.2021.06.002)
Supplement: Document S1. Figures S1–S5, Table S1, Table S3, Table S13, and Table S15 [file mmc1.pdf]

The American Journal of Human Genetics, Volume *108*

Supplemental information

Common haplotypes at the *CFH* locus and low-frequency variants in *CFHR2* and *CFHR5* associate with systemic FHR concentrations and age-related macular degeneration

Laura Lorés-Motta, Anna E. van Beek, Esther Willems, Judith Zandstra, Gerard van Mierlo, Alfred Einhaus, Jean-Luc Mary, Corinne Stucki, Bjorn Bakker, Carel B. Hoyng, Sascha Fauser, Simon J. Clark, Marien I. de Jonge, Everson Nogoceke, Elod Koertvely, Ilse Jongerius, Taco W. Kuijpers, and Anneke I. den Hollander

Supplemental Figures

Figure S1. Correlation matrix of FH, total FHR-1, total FHR-2, FHR-3, FHR-4A and FHR-5 levels in controls and individuals with AMD

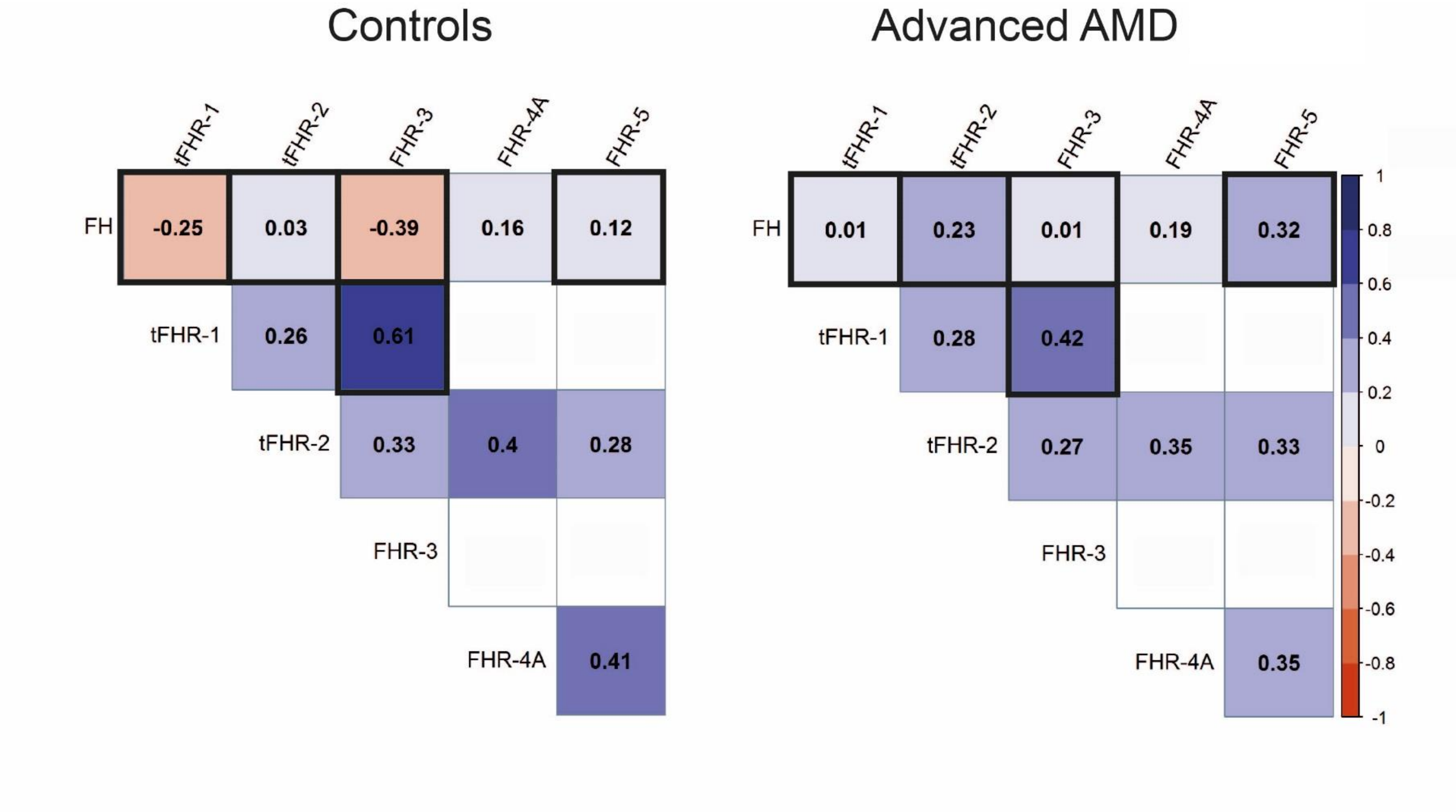

Pearson correlations of log transformed and standardized variables were carried out. Correlation coefficient for correlations with a with a p-value<0.05 are depicted. The colour scale indicates the direction and magnitude of the correlation coefficient. Highlighted squares indicate correlations that are significantly different between controls and individuals with advanced AMD (P-value<0.05).

Correlation analysis of FH and FHR levels revealed significant correlations between these proteins, and some correlations differed between the AMD and the control group. In both the advanced AMD and the control group, total FHR-1 and FHR-3 were correlated with each other, with FH and total FHR-2. Total FHR-2 was correlated with FH and with all other FHR proteins, whereas FHR-4A and FHR-5 were only correlated with FH, with total FHR-2 and with each other. The correlation of total FHR-1 and FHR-3 was much stronger in the control group than in the advanced AMD group (P-value difference= 7.00×10^{-3}). The correlations of FH with total FHR-1, total FHR-2, FHR-3 and FHR-5 also differed between advanced AMD and controls: the correlations with total FHR-1 and FHR-3 were negative in the control group and positive in the advanced AMD group (P-value difference= 8.23×10^{-3} for total FHR-1 and P-value difference=1.29×10^{-5} for FHR-3), and the degree of correlation with total FHR-2 and FHR-5 was higher in the advanced AMD group than in the control group (P-value difference=0.042 for total FHR-2 and P-value difference=0.034 for FHR-5).

Figure S2. Top associations of AMD-associated variants with FHR levels

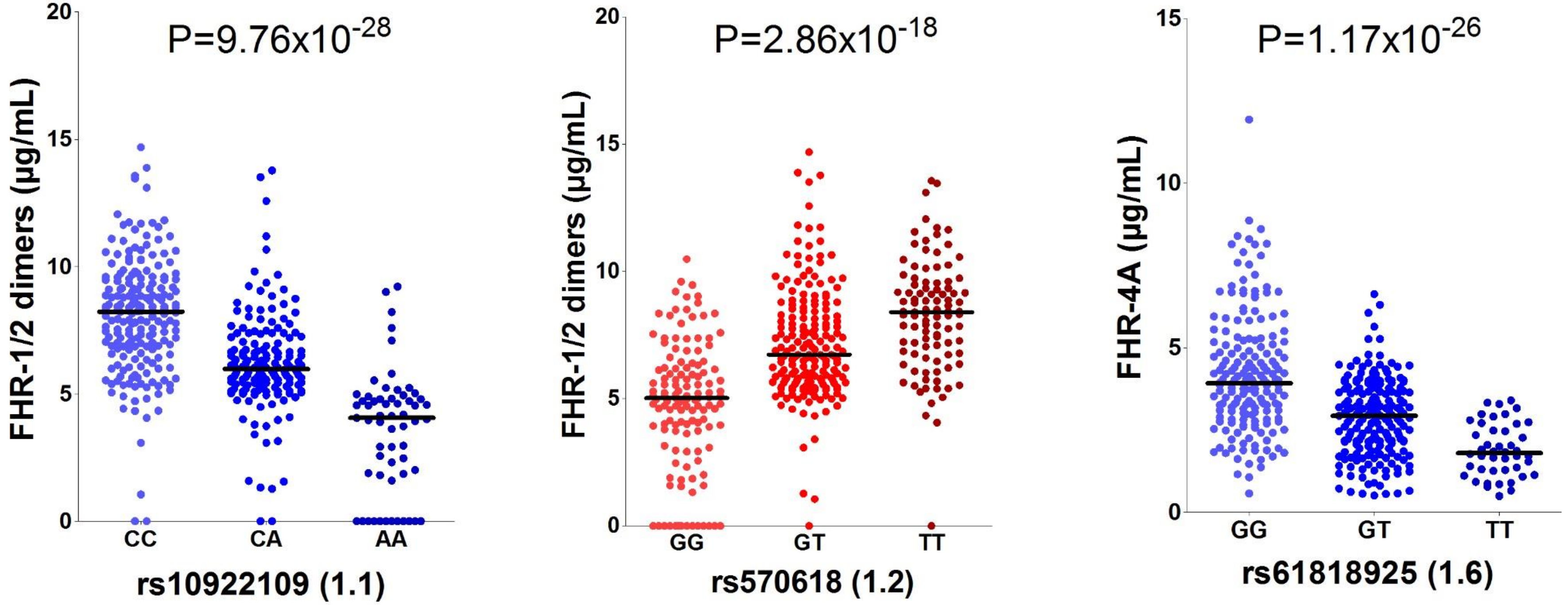

Shown P-values are unadjusted (see Supplemental Table 4 for FDR adjusted P-values). The median value is depicted in each group.

Figure S3. Associations of systemic FH, FHR-1, FHR-2, FHR-3, FHR-4A and FHR-5 with AMD haplotypes at the *CFH* locus

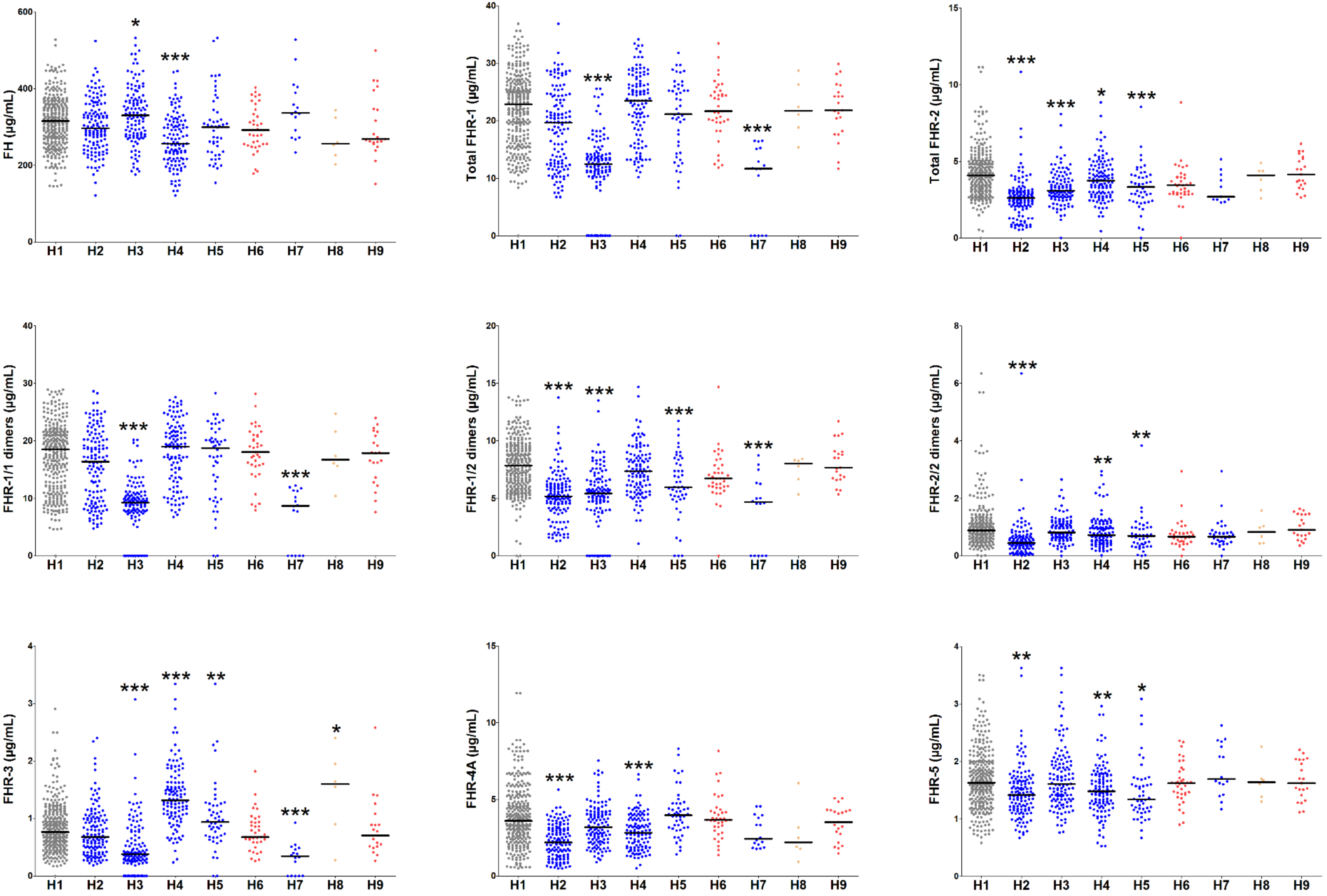
FH (μg/mL)
0
200
400
600
H1 H2 H3 H4 H5 H6 H7 H8 H9
*
***
Total FHR-1 (μg/mL)
0
10
20
30
40
H1 H2 H3 H4 H5 H6 H7 H8 H9
***
***
Total FHR-2 (μg/mL)
0
5
10
15
H1 H2 H3 H4 H5 H6 H7 H8 H9
***
***
*
***
FHR-1/1 dimers (μg/mL)
0
10
20
30
40
H1 H2 H3 H4 H5 H6 H7 H8 H9
***
***
FHR-1/2 dimers (μg/mL)
0
5
10
15
20
H1 H2 H3 H4 H5 H6 H7 H8 H9
***
***
***
***
FHR-2/2 dimers (μg/mL)
0
2
4
6
8
H1 H2 H3 H4 H5 H6 H7 H8 H9
***
**
**
FHR-3 (μg/mL)
0
1
2
3
4
H1 H2 H3 H4 H5 H6 H7 H8 H9
***
***
**
***
*
FHR-4A (μg/mL)
0
5
10
15
H1 H2 H3 H4 H5 H6 H7 H8 H9
***
***
FHR-5 (μg/mL)
0
1
2
3
4
H1 H2 H3 H4 H5 H6 H7 H8 H9
**
**
*

A total of 836 haplotypes from 202 controls and 216 individuals with advanced AMD (highest posterior probability) are included. The haplotype in grey is the reference haplotype, haplotypes in blue are protective for advanced AMD, haplotypes in red are risk-conferring for advanced AMD and the yellow haplotype is not associated with advanced AMD (see Table 2). If a P-value (FDR adjusted) is lower than 0.05, it is represented with one star (*), if it is lower than 0.01, with two (**), and if it is lower than 0.001, with three stars (***). The median value is depicted in each group.

Figure S4. Quantil-quantil plots for the GWAS on FH and FHR levels

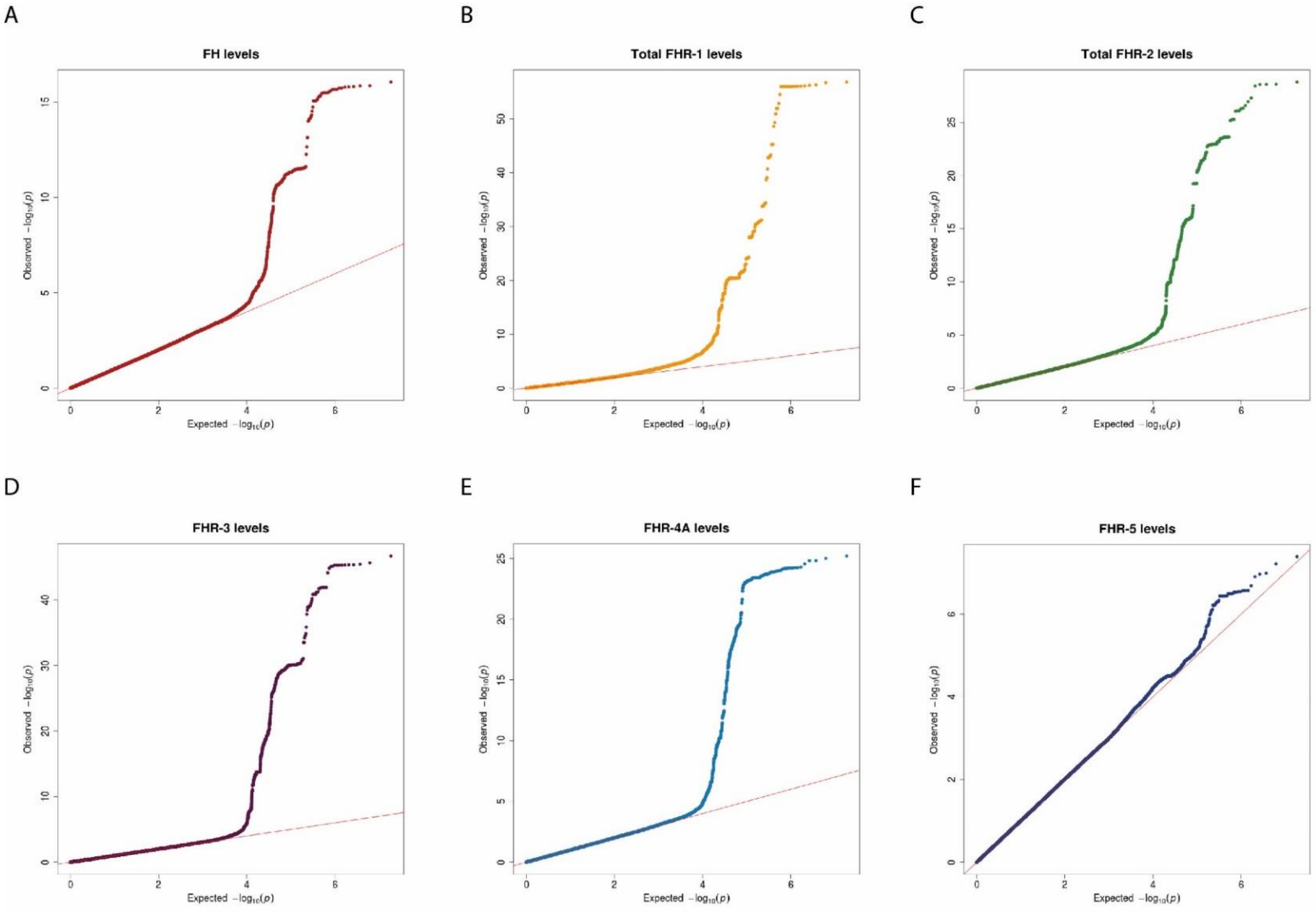

Q-Q plot for the performed GWASes: FH (A), total FHR-1 (B), total FHR-2 (C), FHR-3 (D), FHR-4A (E) and FHR-5 (F). The observed P-values (y axis) are compared to those expected under the null hypothesis of no association (red line, x axis)

Figure S5. Detection of FHR-2 protein using a monospecific antibody

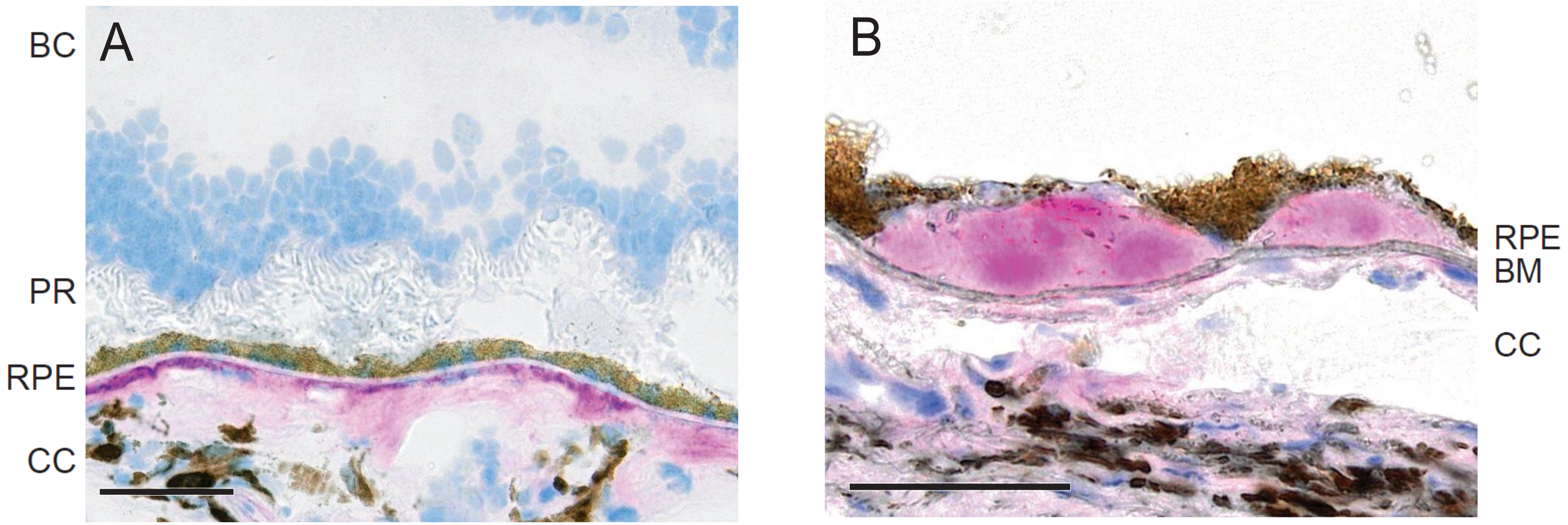

BC=bipoloar cells; PR=photoreceptors; RPE=retinal pigment epithelium; CC=choriocapillaris; BM=Bruch's membrane.

Immunostaining using a monoclonal antibody found to recognize exclusively FHR-2 (R&D Systems, MAB5484). The immunostaining was detected beneath the Bruch's membrane within the intercapillary septa and in the choroid (A). Drusen were also strongly immunopositive (B). Scale bar: 50 μm.

Supplemental Tables

Table S1. Descriptive statistics of the study cohort with FH and FHR measurements

	Advanced AMD	Controls
**n, %**	216 (52.7)	202 (48.3)
**Age mean (SD)**	79.4 (8.5)	71.59 (6.3)
**Female sex (n, %)**	125 (58)	111 (55)
**First batch (n, %)**	72 (33.3)	58 (28.7)
**Cohort - University hospital of Cologne (n, %)**	175 (81.0)	149 (73.8)
**rs187328863 T (MAF)**	0.065	0.03
**rs148553336 C (MAF)**	0.002	0
**rs570618 T (MAF)**	0.593	0.327
**rs10922109 A (MAF)**	0.202	0.478
**rs35292876 T (MAF)**	0.044	0.005
**rs121913059 T (MAF)**	0	0
**rs61818925 T (MAF)**	0.291	0.381
**rs191281603 G (MAF)**	0.007	0.007

N=number; SD=Standard deviation; MAF=minor allele frequency

Table S3. Systemic FHR-1, FHR-2, FHR-3 and FHR-4A levels are elevated in individuals with advanced AMD compared to controls

Protein levels	OR	95% CI		P-value	P-value$_{FDR}$
**FH**	0.979	0.786	1.218	0.847	0.847
**Total FHR-1**	2.136	1.564	2.918	1.84x10-6	5.52x10-6
**Total FHR-2**	1.654	1.276	2.145	1.47x10-4	2.64x10-4
**FHR-1/1**	1.990	1.501	2.638	1.70x10-6	5.52x10-6
**FHR-1/2**	2.043	1.531	2.725	1.18x10-6	5.52x10-6
**FHR-2/2**	1.331	1.042	1.701	0.022	0.028
**FHR-3**	1.692	1.339	2.137	1.05x10-5	2.36x10-5
**FHR-4A**	1.325	1.063	1.651	0.012	0.018
**FHR-5**	1.260	0.998	1.592	0.052	0.058

Association analysis of FH and FHR levels with advanced AMD was performed by means of a logistic regression adjusted for age, sex, measuring batch and sampling cohort. The resulting odds ratios (OR) reflect the change in odds for advanced AMD per 1-standard deviation increase of each log-transformed +1 protein levels. Correction for multiple testing was performed by applying the Benjamini-Hochberg procedure, a false discovery rate (FDR) correction, due to a

strong correlation between the proteins and the dimers being derived measurements. The threshold for statistical significance was defined as FDR adjusted P-value <0.05.

Table S13. Sensitivity analysis of the gene-based tests adjusting for ten ancestry principal components confirms the association of low frequency and rare variants in CFH, CFHR-2 and CFHR-5 with age-related macular degeneration

Gene	Chromosomal location[a]	N of variants included	N of singletons included	N of individuals carrying low frequency and rare variants	AC in AMD	AC in controls	P-value	SKAT-O ρ
CFH	1:196621254-196716353	55	13	2077	922	1364	$5.19x10^{-6}$	0
CFHR1	1:196794703-196797262	4	2	11	6	5	0.598	1
CFHR2	1:196918615-196928188	11	3	3177	1262	2026	$5.43x10^{-3}$	0.3
CFHR3	1:196748349-196748468	4	2	31	15	16	0.587	0
CFHR4	1:196871563-196887530	20	1	555	191	378	0.382	0
CFHR5	1:196952046-196977807	30	10	2771	1247	1708	$1.42x10^{-6}$	0

AC=allele count

[a] Chromosomal position according to the NCBI RefSeq hg19 human genome reference assembly.

SKAT-O gene based tests were carried out to compare protein altering variants with a minor allele frequency <0.05 in 17.596 controls and 15.894 individuals with AMD (a total of 33.488 individuals). The STAK-O tests were adjusted for the first ten ancestry principal components, source of DNA (whole-blood or whole-genome amplified DNA) and variants in the locus that have been previously reported to be associated with AMD (rs187328863, rs148553336, rs570618, rs10922109, rs35292876, rs121913059, rs61818925 and rs191281603, Fritsche et al., 2015). Details about the variants included in the test are displayed in Supplemental table 14.

Table S15. Gene-based tests results including variants with a CADD PHRED score >10

Gene	Chromosomal location[a]	N of variants included	N of singletons included	N of individuals carrying low frequency and rare variants	AC in AMD	AC in controls	P-value	SKAT-O ρ
CFH	1:196621254-196716353	40	12	1721	610	1138	2.77×10^{-6}	0
CFHR1	1:196794703-196797262	4	2	11	6	5	0.601	1
CFHR2	1:196918615-196928188	8	3	2785	1123	1744	3.26×10^{-3}	0.4
CFHR3	1:196748349-196748468	3	2	24	14	10	0.492	0
CFHR4	1:196871563-196887530	11	1	474	160	318	0.340	0
CFHR5	1:196952046-196977807	19	6	2122	835	1410	8.62×10^{-7}	0.2

AC=allele count

[a] Chromosomal position according to the NCBI RefSeq hg19 human genome reference assembly.

SKAT-O gene based tests were carried out to compare protein altering variants with a CADD score ≥10 and a minor allele frequency <0.05 in 17.596 controls and 15.894 individuals with AMD (a total of 33.488 individuals). PHRED-scaled CADD scores were assigned using CADD v1.4 (see Supplemental Table 14 for the individual variants scores). Variants with PHRED-scaled CADD scores higher than 10 are predicted to be the 10% most deleterious substitutions expected

in the human genome. The STAK-O tests were adjusted for the first two ancestry principal components, source of DNA (whole-blood or whole-genome amplified DNA) and variants in the locus that have been previously reported to be associated with AMD (rs187328863, rs148553336, rs570618, rs10922109, rs35292876, rs121913059, rs61818925 and rs191281603, Fritsche et al., 2015). Details about the variants included in the test are displayed in Supplemental table 14.

Table S16. Low-frequency variants in *CFHR2* and *CFHR5* are associated with reduced FHR-2 and FHR-5 levels

Protein levels	Gene	Variant	Allele count	B	SE	P-value	$P\text{-value}_{FDR}$
**FH**	*CFH*	FH p.Asn1050Tyr	18	0.130	0.235	0.580	0.695
**FH**	*CFH*	FH p.Gln950His	7	0.664	0.359	0.065	0.156
**FH**	*CFH*	FH p.Gly650Val	2	0.826	0.658	0.210	0.420
**FH**	*CFH*	FH p.Thr956Met	2	-0.618	0.684	0.367	0.551
**Total FHR-2**	*CFHR2*	FHR-2 p.Cys72Tyr	10	-2.334	0.272	2.46X10-16	2.95X10-15
**Total FHR-2**	*CFHR2*	FHR-2 p.Glu199Ter	10	-1.491	0.266	4.04X10-8	2.42X10-7
**Total FHR-2**	*CFHR2*	FHR-2 p.Tyr264Cys	7	-1.524	0.327	4.31X10-6	1.72X10-5
**Total FHR-2**	*CFHR2*	FHR-2 p.Thr109Ala	4	-0.188	0.429	0.662	0.695
**Total FHR-2**	*CFHR2*	FHR-2 p.His30Arg	2	-0.420	0.605	0.488	0.651
**FHR-5**	*CFHR5*	FHR-5 p.Arg356His	13	0.265	0.279	0.344	0.551
**FHR-5**	*CFHR5*	FHR-5 p.Pro46Ser	5	-0.171	0.436	0.695	0.695
**FHR-5**	*CFHR5*	FHR-5 p.Cys208Arg	2	-1.869	0.679	0.006	0.018

Association analysis of low-frequency and rare variants in *CFH*, *CFHR2* and *CFHR5*, with protein measurements of FH, FHR-2 and FHR-5 respectively. Analyses were adjusted for age, sex, measuring batch, sampling cohort, AMD status and the variants in the locus that have been previously reported to be associated with AMD (rs187328863, rs148553336, rs570618, rs10922109, rs35292876, rs121913059, rs61818925 and rs191281603; Fritsche et al., 2015). The resulting betas reflect the variant effect on log-transformed +1 and standardized protein levels.
